# Supplementary material for: Optimization of ACE-tRNAs function in translation for suppression of nonsense mutations
Source: Nucleic Acids Res. 2024 Nov 30;52(22):14112–32. doi: 10.1093/nar/gkae1112 (PMC11662937; doi:10.1093/nar/gkae1112)
Supplement: gkae1112_Supplemental_Files [file gkae1112_supplemental_files.zip › Supplemental Information_FINAL.docx]

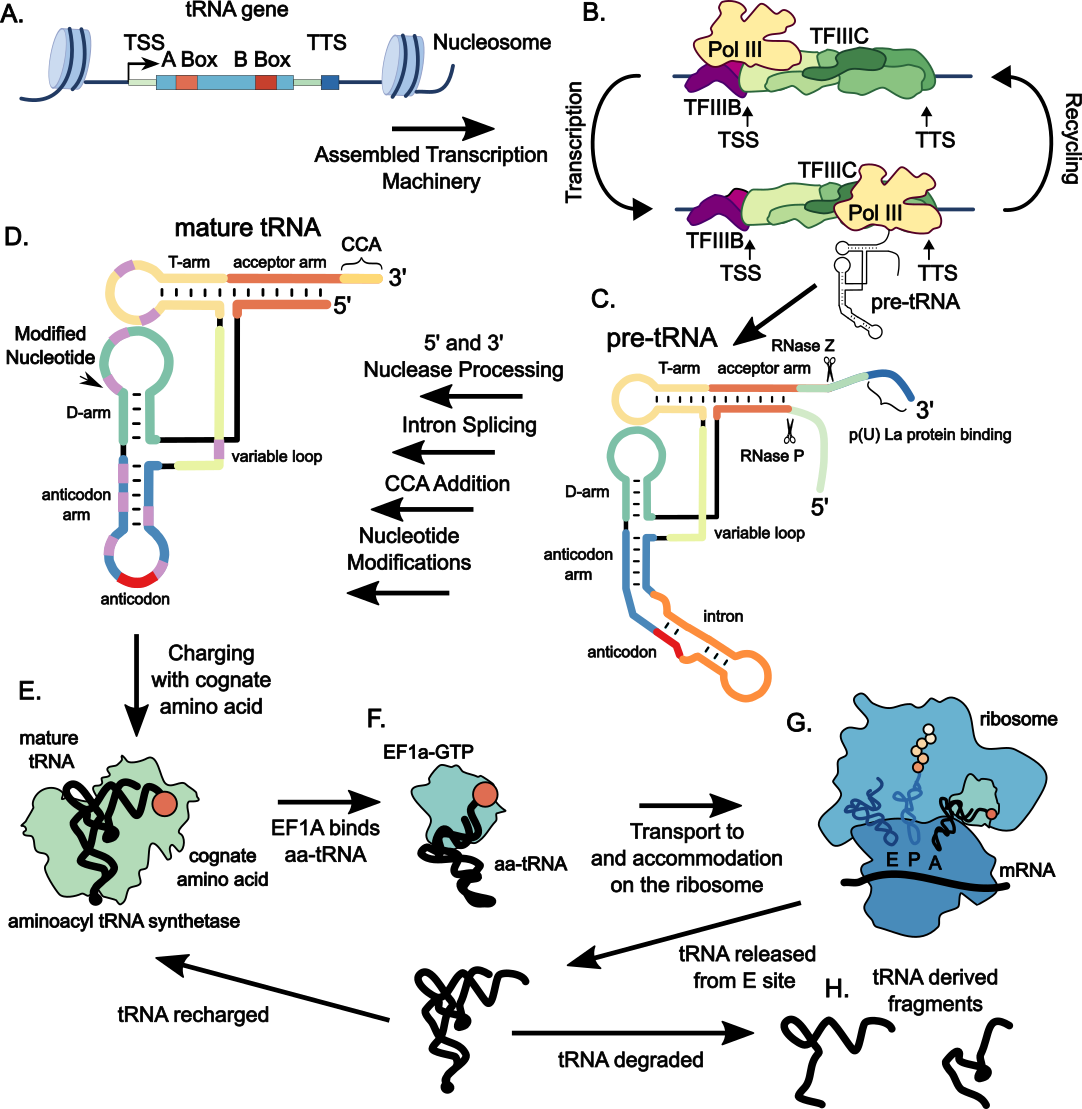


**Supplemental Figure 1.** **Natural tRNA biogenesis and function in translation** (**A**) The human genome contains ~500 tDNAs^1^, which are subjected to epigenetic regulation of expression. While nearly half of human tDNAs are silent^2^, actively expressed tDNAs display nucleosome-free gene cassettes, delimited by nucleosomes centered ~150 bp upstream of the transcription start site (TSS) and ~125 bp downstream of the transcription termination signal (TTS)^3^. (**B**) tDNA located in areas of active chromatin are transcribed by RNA polymerase III (Pol III). tRNAs are expressed from type 2 Pol III promoters with the multi-subunit transcription factor III C (TFIIIC) binding to A and B box sequences (orange and red boxes in panel A) which are internal to the mature tRNA sequence (light blue box in panel A)^4-6^. TFIIIC then recruits the multi-subunit transcription factor IIIB (TFIIIB) to the tRNA upstream control element sequence ~50 bp upstream of the tRNA which contains a weak TATA box and the TSS^7^. TFIIIB then recruits RNA Pol III which transcribes the pre-tRNA. Following formation of the transcription complex multiple rounds of transcription take place before the factors dissociate from the tRNA gene^8^. The TTS consists of a stretch of greater than four (in humans) thymidines^4^. (**C**) The pre-tRNA transcript is synthesized with 5’-leader and 3’-trailer sequences at either end. The short polyU tract remaining at the 3’ end of the pre-tRNA serves as the binding site for the La protein, which protects the 3’ end of the transcript from spurious exonuclease digestion and helps with pre-tRNA folding^9^. (**D**) The 5’-leader and 3’-trailer are removed through the endonuclease action of the ribonucleoprotein RNase P (5’ end) and RNase Z (3’ end), along with the action of other exonucleases^10-12^. Tuning has been demonstrated in pre-tRNA binding to RNase P between the 5’ leader sequence in the pre-tRNA and the tRNA body^13^. Following 3’ cleavage, the CCA adding enzyme catalyzes the addition of CCA nucleotides to the tRNA 3’ terminus without the need for a template^14^. A subset of tRNAs contain introns which are primarily located immediately 3’ to the anticodon. As these tRNA introns disrupt the anticodon arm, they must be removed via splicing to produce a functional mature tRNA^15^. Following processing, ~12% of tRNA nucleotides are modified with one of many modifications, both before and after trafficking from the nucleus to the cytoplasm^16,17^. tRNA chemical modifications play a role in all aspects of tRNA function including, structure, stability, aminoacylation, and decoding at the ribosome^17^. (**E**) Mature tRNAs interact with their cognate aminoacyl-tRNA synthetase (aaRS) and are charged with their cognate amino acid^18,19^. Recognition by the correct aaRS represents the first major step in maintaining translational fidelity, along with ensuring the correct codon-anticodon pairing on the ribosome. (**F**) The aminoacyl tRNA (aa-tRNA) is then passed to GTP-bound translation elongation factor 1a (EF1a), which shuttles the aa-tRNA to the ribosomal A site, forming a ribosome-EF1a-aa-tRNA ternary complex^20,21^. Sequences in the t-stem of tRNAs are primarily responsible for tuning affinities for EF1a^22,23^. (**G**) If the codon-anticodon interaction is correct, the EF1a-bound GTP is hydrolyzed, and EF1a releases the aa-tRNA, which transits the ribosome extending the polypeptide chain in protein synthesis^24^. Binding of the aa-tRNA with EF1a to the A site of the ribosome and interactions with the mRNA occurs in discrete steps that require flexibility in the tRNA structure^25-27^. (**H**) While tRNAs are generally stable, exhibiting a half-life of 2-3 days in eukaryotes^28^, their steady state level is influenced by complex turnover mechanisms^29^. tRNA derived fragments, formed following tRNA breakdown have been increasingly shown to influence biology. Given the importance of RNA fragments in a number of aspect of gene regulation, the emerging role of tRNA fragments in development and disease is not surprising^30,31^.


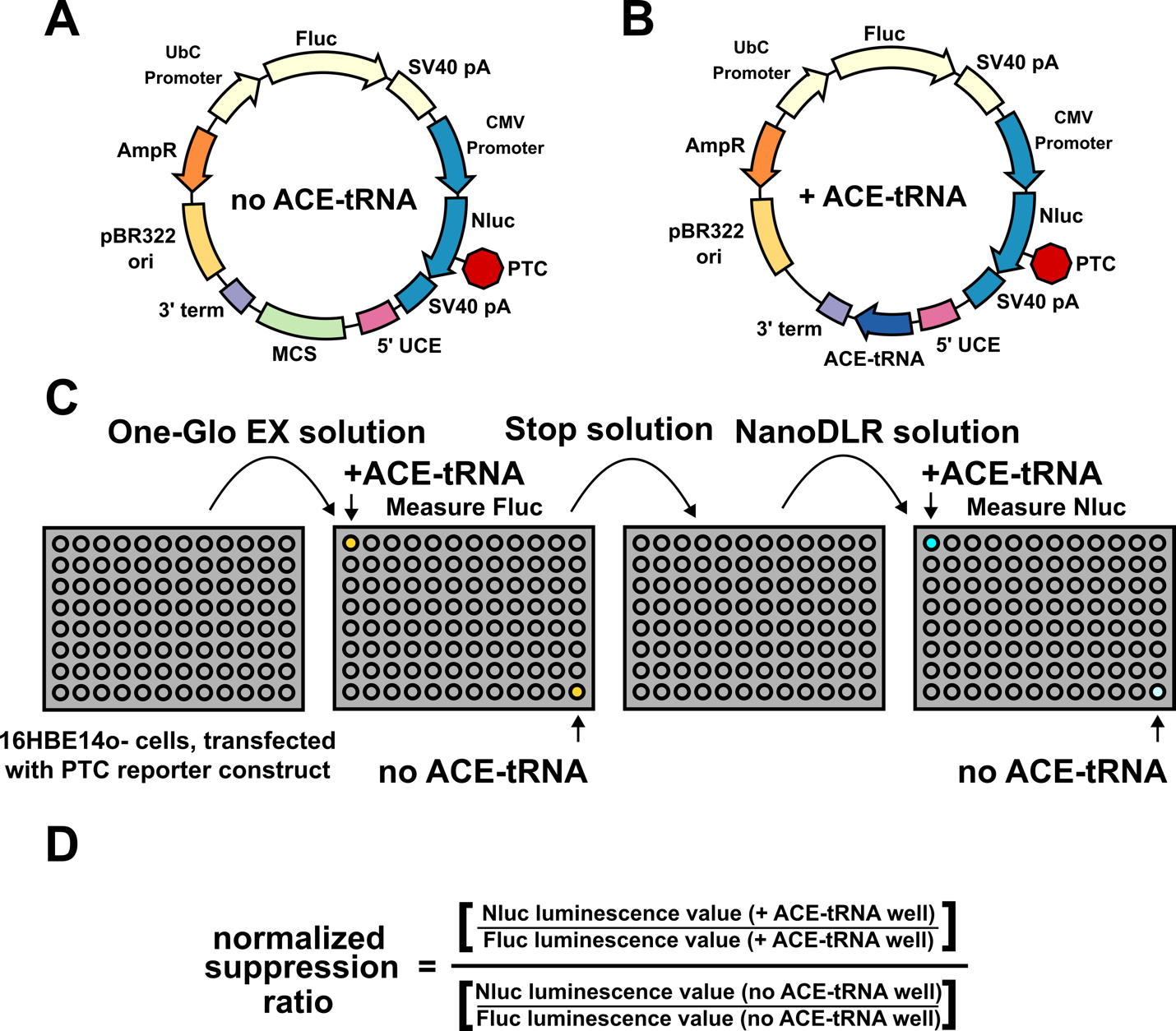


**Supplemental Figure 2.** **PTC reporter constructs and PTC suppression assay scheme** (**A**) The pNanoRePorter 2.0 PTC reporter plasmid contains a firefly luciferase (Fluc) expression cassette for transfection normalization, a PTC-interrupted nanoluciferase (Nluc) expression cassette for high-throughput readout of PTC suppression, and a multiple cloning site (MCS) containing a ccdB negative selection marker for high-throughput cloning of ACE-tRNA sequences. The construct containing the MCS is used as a negative control as it contains no ACE-tRNA and thus only reports on basal cellular PTC suppression. (**B**) Displays a generic ACE-tRNA-containing pNanoRePorter 2.0 construct, which is a stand-in for any of the >1,800 ACE-tRNA expression cassette sequences tested here. (**C**) Following transfection of construct A (lower right well) and construct B (upper left well) into 16HBE14o- cells in 96-well format, the plates were maintained in a CO_2_ cell culture incubator for 24 hours. After incubation, the media was aspirated, ONE-Glo Ex solution (Nano-Glo Dual-Luciferase Assay Reporter Kit, Promega) containing Fluc substrate was added to the wells, and Fluc luminescence of each well was assayed by plate reader. After the Fluc luminescence was assayed, Stop solution was added to each well to quench Fluc luminescence. After Fluc luminescence was quenched, NanoDLR solution was added to each well and the Nluc luminescence was assayed by plate reader. High Nluc luminescence is seen for wells transfected with reporter constructs containing ACE-tRNAs, while low Nluc luminescence is seen for wells transfected with reporter constructs containing no ACE-tRNA. (**D**) The normalized suppression ratio for each construct is computed based on the equation shown here. Every plate contains a well transfected with the “no ACE-tRNA” control to normalize PTC suppression for all wells in that plate to account for basal cellular PTC suppression.

**Supplemental Figure 3.** **Derivation of 5’ upstream control element sequences and results of extragenic screens for HEK293 cells** (**A**) The parent high-throughput-cloning and -screening vector for the tRNA 5’ region, contains a cloning site immediately 5’ to the original ArgUGA (oArgUGA) tRNA sequence/the original 3’ trailer sequence. (**B**) All human tRNAs are denoted with a name like “tRNA-Ala-AGC-1-1” (where Ala is the three-letter amino acid code for the tRNA isotype, AGC is the anticodon, the first 1 corresponds to the numeric ID of a unique tRNA transcript or “isodecoder”, and the second 1 corresponds to the gene locus ID – for tRNAs that have multiple identical copies, this gene locus ID represents the particular gene copy in the genome). Scanning the entire human genome returns 386 predicted tRNAs with a unique 55 bp sequence immediately upstream of the tRNA. (**C)** To determine the impact of these different sequences on the function of ACE-tRNAs we had each unique 55 bp 5’ upstream control element (UCE, what we call this 5’ upstream transcriptional element) synthesized as complementary oligonucleotides, which when annealed provide overhangs for golden gate cloning into our parent vector shown in (A). (**D**) Heat map representing the results of screening a 386-member library of 55-bp 5’ UCE sequences derived from each unique tRNA gene 5’ UCE in the human genome in HEK293 cells. (**E**) Heat map representing the results of screening a 256-member library of 4-bp 3’ trailer sequences representing every 4-bp combination of nucleotides following the ACE-tRNA in HEK293 cells. (**F**) Heat map representing the results of screening a 326-member library of 850-bp 5’ UCE sequences derived from every synthetically accessible, unique, 5’ UCE in the human genome in HEK293 cells. All values displayed in these heat maps represent the average of 6 independent transfections of HTCS library members. The normalized suppression ratio shown here is calculated from the equation (PTC-NanoLuciferase luminescence [+ACE-tRNA]/Firefly luminescence)/(PTC-Nanoluciferase luminescence [no ACE-tRNA]/Firefly luminescence).

**Supplemental Figure 4.** **Comparison of 5’ UCE sequences.** The influence of the 55 bp versus 850 bp 5’ UCE sequences on the nonsense suppression efficiency of ACE-tRNA^Arg^_UGA_ was compared in both 16HBE14o- (**A**) and HEK293T (**B**) cell lines. The line of best fit (y-intercept set to y = 0) for each data set (red line) was determined using Prism 10 (slope = 0.9065 for A and slope = 1.328 for B), with the dotted black line representing a line with a slope of 1. The influence of the 55 bp 5’ UCE sequences were also compared with codon abundance in the human transcriptome for both 16HBE14o- (**C**) and HEK293T (**D**) cell lines. For each 55 bp 5’ UCE, the cognate codon corresponding to the tRNA from which it was derived (Supplemental Fig. 2) was determined, and the percent of all codons in the human transcriptome was determined for each codon. The line of best fit for each data set (red line) was determined using Prism 10 (R^2^ = 0.005455 for C and R^2^ = 0.001112 for D).

**Supplemental Figure 5.** **Types of RNA Pol III promoters and their impact on ACE-tRNA function** (**A**) Type 2 RNA Pol III promoters are employed to expressed tRNA genes in humans. Intragenic box A and box B sequences recruit TFIIIC, which then recruits TFIIIB, which then recruits RNA Pol III. Type 3 RNA Pol III promoters express other genes including U6 and H1 RNAs. Type 3 promoters do not require any intragenic sequences and as such are often used to express exogenous RNAs including CRISPR guide RNAs, siRNAs, and shRNAs which do not contain native A or B boxes. Type 3 promoters have been used to express human nonsense suppressor tRNAs although many of the endogenous human 55 bp 5’-leaders yield higher nonsense suppression activity *in vivo*. (**B**) Each of the 5’ upstream sequences was cloned upstream of ACE-tRNA^Arg^_UGA_ and transfected into 16HBE14o- cells. tRNA-Tyr-GTA-5-1 5’ UCE represents the original 5’ UCE and tRNA-Cys-GCE-12-1 5’ UCE represents the best-performing 5’ UCE obtained from the 55-bp 5’ UCE screen. The normalized suppression ratio shown here is calculated from the equation (PTC-NanoLuciferase luminescence [+ACE-tRNA]/Firefly luminescence)/(PTC-Nanoluciferase luminescence [no ACE-tRNA]/Firefly luminescence). The error bars represent the standard error of the mean.

**Supplemental Figure 6.** **T-stem mutations tested** (**A**) Sequences for each of the t-stem nucleotide pairs 49-65, 50-64, 51-63. These sequences were derived from (Saks et al., 2011), with the variant numbering in the first column representing the number scheme used in Saks et al. and the numbering in the second column representing the numbering scheme used in this paper. (**B**) Depicts a generic t-stem with canonical tRNA nucleotide position numbering.

**Supplemental Figure 7.** **Influence of individual ACE-tRNA^Arg^_UGA_ and ACE-tRNA^Leu^_UGA_ sticky stem sites on nonsense suppressor efficiency.** Sites for each of the sticky stem library pairs are labeled 1-7 on the tRNA diagrams. The heatmap representations of each sticky stem screen are shown below. The presence of a number means that this library member is present in the sequence, while no number means the original pair is present in that sequence.

**Supplemental Figure 8.** **Effect of 5’ UCE sequences on ACE-tRNA^Arg^_UGA_ nonsense suppression activity compared to impact of the same 5’ UCE sequences on steady-state ACE-tRNA^Arg^_UGA_ expression levels** The equation for the line of best fit is displayed on the graph with the R^2^ value


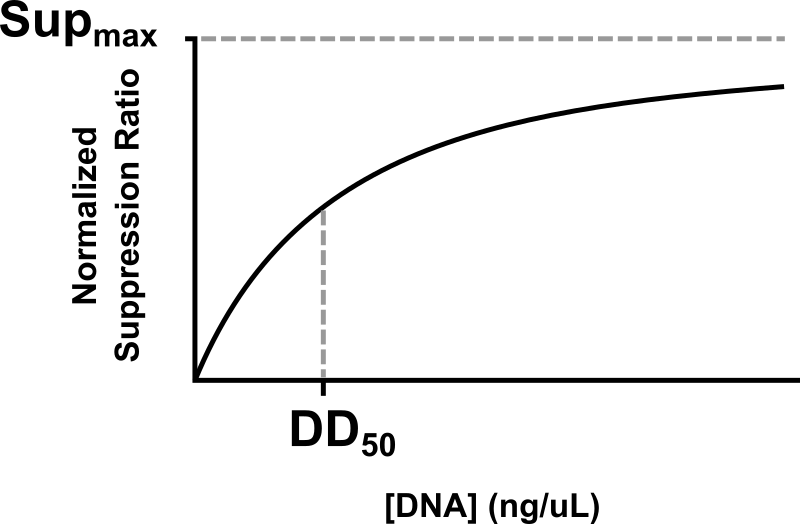


**Supplemental Figure 9.** **Model to fit DNA dependence of ACE-tRNA nonsense suppressor efficiency** This diagram outlines the parameters of the hyperbolic model to fit the DNA concentration dependence of ACE-tRNA nonsense suppression efficiency. The model is defined by the equation Normalized Suppression Ratio = (Sup_max_ * [DNA])/(DD_50_ + [DNA]) where Sup_max_ corresponds to the maximal level of nonsense suppression displayed by the ACE-tRNA and DD_50_ (Delivered DNA) corresponds to the concentration of DNA (ng/μL) required for a half-maximal nonsense suppression response.

**Supplemental Figure 10.** **All sfGFP p.150 amino acid variants are detected by tryptic digest mass spectrometry** (**A**) All 20 variants of sfGFP with each AA at p.150 were cloned in pBad vectors and expressed in *E. coli*. The sfGFP variants were purified using a C-terminal twin strep tag, resolved by SDS-PAGE, and the protein stained with coomassie (**B**) The samples shown in panel A were mixed at equimolar amounts, subjected to in-solution tryptic digest, and the masses of the peptide containing p.150 were determined by mass spectrometry as outlined in the methods section. The measured masses for the b and y ions are shown for the representative peptide containing glycine at p.150 (LEYNFNSH**G**VYITADK) with the spectrum of b and y ion peaks shown in (**C**). (**D**) The p.150 peptide for every amino acid was evident from the MS data of the mixture. The total abundance (sum of correct cleavage and 1 missed cleavage) of the p.150 peptide of interest was calculated and the relative peptide abundance was calculated for every peptide abundance normalized to the lowest abundance.

**Supplemental Figure 11.** **Uncropped gel images of gels shown in Figure 6B** (**A**) Alexa 488 fluorescence image of the crude 10-20% gradient SDS-PAGE gel (Invitrogen) of HEK293 cell lysate containing WT- or sfGFP-TGA-150 (**B**) subsequent silver staining of the crude SDS-PAGE gel.

**Supplemental Figure 12.** **Purification of sfGFP from HEK293 cells** A construct expressing sfGFP-UGA-150-Strep-8xHis-Strep with a construct expressing 4 copies of the ACE-tRNA as noted above were co-transfected into HEK293 cells. 48 hours after transfection the cells were harvested, the soluble protein extracted via dounce homogenization, the lysate clarified via centrifugation (30 min at 25k rcf) followed by filtration (0.45 μm filter). The clarified cell lystate was allowed to flow through a Strep-Tactin XT Superflow column and the flowthrough was collected for analysis. The bound protein was washed extensively with wash buffer (100 mM Tris-HCl pH 8.0, 150 mM NaCl, 1 mM EDTA) and eluted in wash buffer containing 50 mM d-biotin. The protein samples were resolved on a 10-20% gradient SDS-PAGE gel (Invitrogen) and stained with the Pierce Silver Stain for MS kit (Invitrogen).

**Supplemental Figure 13.** **Uncropped gel images of gels shown in Figure 6C**

**Supplemental Figure 14.** **Uncropped Nluc in-gel luminescence images of gels shown in Figure 7B**

**
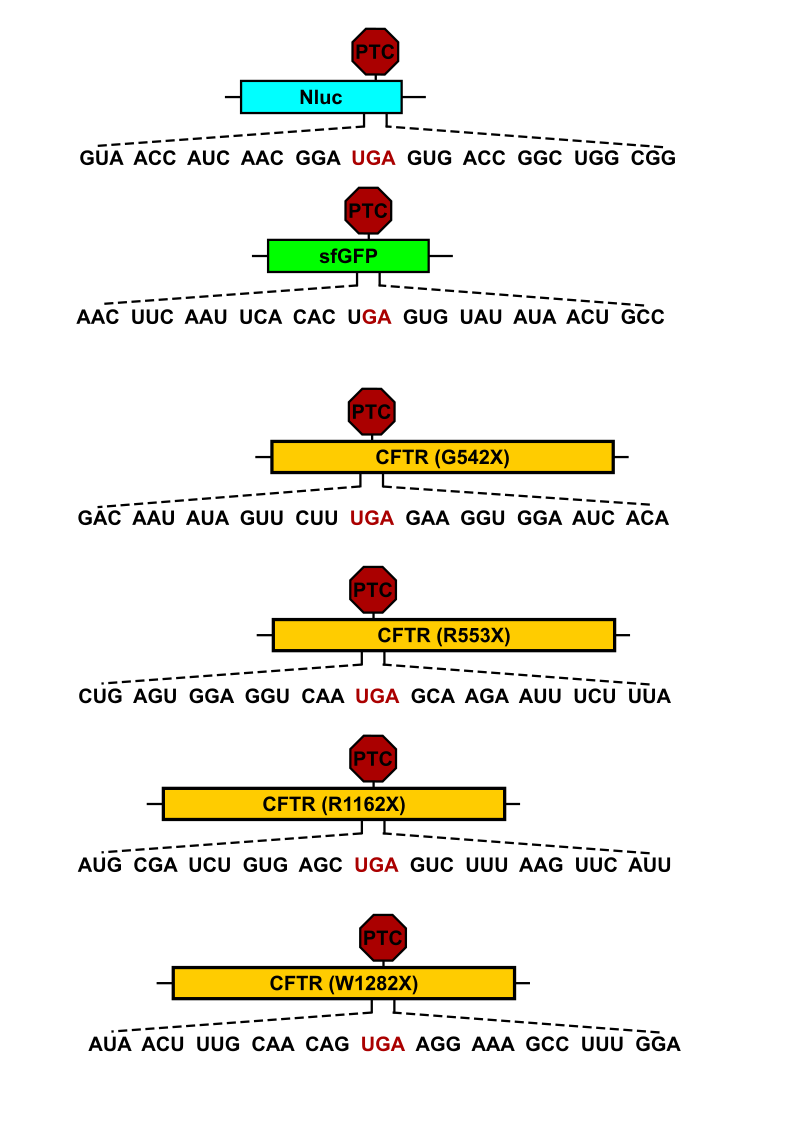
**

**Supplemental Figure 15.** **mRNA contexts of reporter PTCs used in this study**

**
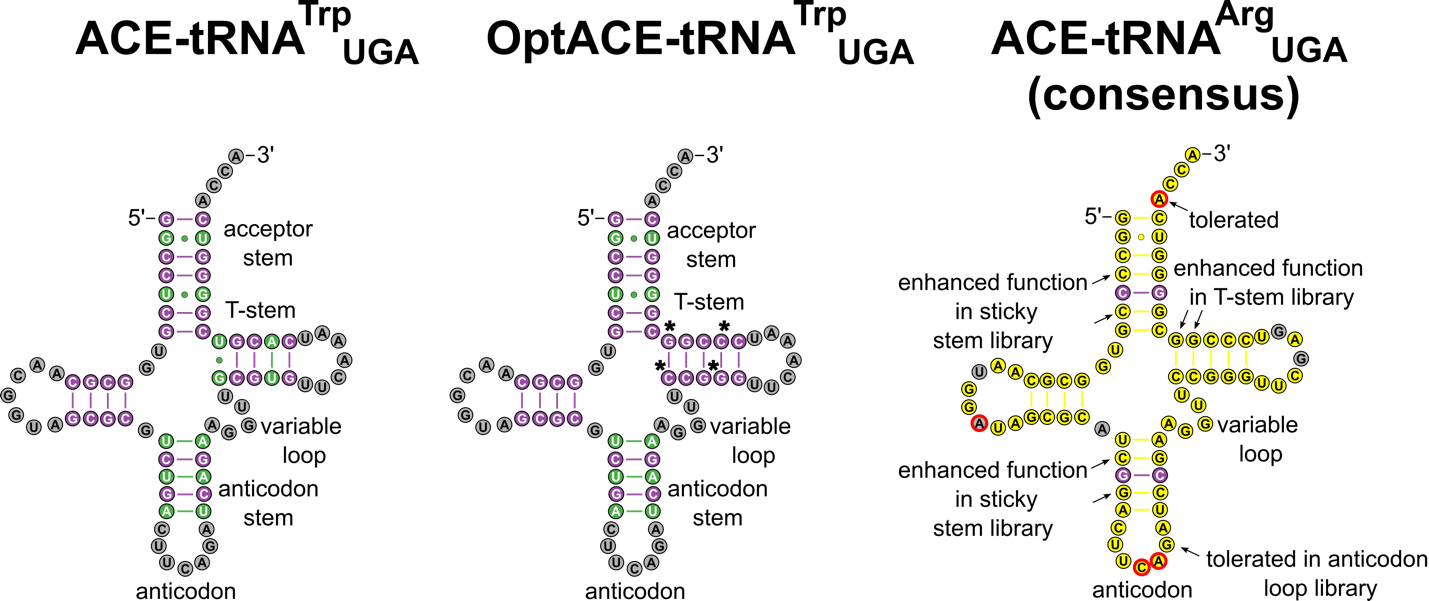
**

**Supplemental Figure 16.** **Comparison of OptACE-tRNA^Trp^_UGA_** **and ACE-tRNA^Arg^_UGA_** Shown are cloverleaf models of ACE-tRNAs, with substitutions to the t-stem as compared to the original ACE-tRNA^Trp^_UGA_ denoted with * for OptACE-tRNA^Trp^_UGA_. A consensus sequence is presented for ACE-tRNA^Arg^_UGA_ with allowed substitutions denoted with arrows as demonstrated in screens conducted for this study or known to exist in human tRNA^Arg^ sequences. Nucleotides highlighted in yellow indicate common nucleotide identities when comparing OptACE-tRNA^Trp^_UGA_ and ACE-tRNA^Arg^_UGA_. Nucleotides with red outlines denote strong identity elements for tRNA^Arg^ as described in^32^.

| ACE-tRNA expression cassette | Sup_max_ (arbitrary units) | DD_50_ (ng/μL) |
| --- | --- | --- |
| Original ArgUGA | 4000 ± 150 | 11.3 ± 1.2 |
| Optimized ArgUGA | 5300 ± 150 | 5.0 ± 0.6 |
| Original LeuUGA | 1300 ± 30 | 5.6 ± 0.4 |
| Optimized LeuUGA | 1330 ± 35 | 1.6 ± 0.2 |
| Original GlyUGA | 560 ± 30 | 23 ± 3 |
| Optimized GlyUGA | 590 ± 35 | 16 ± 2 |
| Original TrpUGA | 66 ± 4 | 23 ± 3 |
| Optimized TrpUGA | 240 ± 20 | 24 ± 5 |

Supplemental Table 1. **Fit data for plots displayed in Figure 5.** Curves were fit as outlined in the legend for Supplemental Figure 7.

|  | **Arg (%)** | **Asn (%)** | **Leu (%)** | **Gly (%)** |
| --- | --- | --- | --- | --- |
| Original ArgUGA | 99.46 | 0.54 | 0.00 | 0.00 |
| Optimized ArgUGA | 99.85 | 0.15 | 0.00 | 0.00 |
| Original LeuUGA | 0.00 | 2.25 | 97.75 | 0.00 |
| Optimized LeuUGA | 0.19 | 0.45 | 99.29 | 0.07 |
| Optimized GlyUGA  Optimized TrpUGA | 0.90  99.99 | 1.92  0.01 | 0.00  0.00 | 97.18  0.00 |
| Weighted Original ArgUGA | 99.24 | 0.76 | 0.00 | 0.00 |
| Weighted Optimized ArgUGA | 99.80 | 0.20 | 0.00 | 0.00 |
| Weighted Original LeuUGA | 0.00 | 1.45 | 98.55 | 0.00 |
| Weighted Optimized LeuUGA | 0.09 | 0.29 | 99.57 | 0.05 |
| Weighted Optimized GlyUGA | 0.51 | 1.52 | 0.00 | 98.0 |
| Weighted Optimized TrpUGA | 99.99 | 0.01 | 0.00 | 0.00 |

Supplemental Table 2. **Percent of each amino acid incorporated at position 150 in sfGFP-TGA-150 as determined by mass spectrometry.** Percent values of incorporation for each amino acid for data shown in Figure 6. Weighted values were calculated using the relative abundances for every amino acid from the mixed sfGFP MS control sample to account for any differences in trypsinization of ionization efficiency of the peptides.

**Genbank sequence for 5’ HTCS vector**

LOCUS 5prime_pNanoRePo 7167 bp DNA circular SYN 29-MAR-2023

DEFINITION synthetic circular DNA

ACCESSION .

VERSION .

KEYWORDS .

SOURCE synthetic DNA construct

ORGANISM synthetic DNA construct

REFERENCE 1 (bases 1 to 7167)

AUTHORS .

TITLE Direct Submission

JOURNAL Exported Mar 29, 2023 from SnapGene 6.2.1

https://www.snapgene.com

COMMENT LOCUS pcDNA3.1/Zeo(+) 5015 bp DNA SYN

DEFINITION pcDNA3.1/Zeo(+)

ACCESSION

KEYWORDS

SOURCE

ORGANISM other sequences; artificial sequences; vectors.

FEATURES Location/Qualifiers

source 1..7167

/mol_type="other DNA"

/organism="synthetic DNA construct"

source 5253..5325

/lab_host="E.coli"

/db_xref="taxon:66198"

/organism="Cloning vector pUC57"

promoter 36..435

/label=UbC promoter

/note="human ubiquitin C promoter"

promoter 445..463

/label=T7 promoter

/note="promoter for bacteriophage T7 RNA polymerase"

regulatory 491..500

/label=Kozak sequence

/note="vertebrate consensus sequence for strong initiation

of translation (Kozak, 1987)"

/regulatory_class="other"

CDS 497..2146

/codon_start=1

/product="firefly luciferase reporter gene luc2 (Photinus

pyralis) and is designed for high expression and reduced

anomalous transcription. This sequence was engineered with

fewer consensus regulatory sequences and has been codon

optimized for mammalian expression."

/label=Fluc2 (From Promega pGL4.10[luc2] vector)

/note="synthetic luc2 version of the luciferase gene"

/translation="MEDAKNIKKGPAPFYPLEDGTAGEQLHKAMKRYALVPGTIAFTDA

HIEVDITYAEYFEMSVRLAEAMKRYGLNTNHRIVVCSENSLQFFMPVLGALFIGVAVAP

ANDIYNERELLNSMGISQPTVVFVSKKGLQKILNVQKKLPIIQKIIIMDSKTDYQGFQS

MYTFVTSHLPPGFNEYDFVPESFDRDKTIALIMNSSGSTGLPKGVALPHRTACVRFSHA

RDPIFGNQIIPDTAILSVVPFHHGFGMFTTLGYLICGFRVVLMYRFEEELFLRSLQDYK

IQSALLVPTLFSFFAKSTLIDKYDLSNLHEIASGGAPLSKEVGEAVAKRFHLPGIRQGY

GLTETTSAILITPEGDDKPGAVGKVVPFFEAKVVDLDTGKTLGVNQRGELCVRGPMIMS

GYVNNPEATNALIDKDGWLHSGDIAYWDEDEHFFIVDRLKSLIKYKGYQVAPAELESIL

LQHPNIFDAGVAGLPDDDAGELPAAVVVLEHGKTMTEKEIVDYVASQVTTAKKLRGGVV

FVDEVPKGLTGKLDARKIREILIKAKKGGKIAV"

misc_feature 2162..2281

/label=SV40 poly(A) signal

enhancer 2351..2730

/label=CMV immediate early enhancer

/note="human cytomegalovirus immediate early enhancer"

promoter 2731..2934

/label=CMV promoter

/note="human cytomegalovirus (CMV) immediate early

promoter"

promoter 2979..2997

/label=T7 promoter

/note="promoter for bacteriophage T7 RNA polymerase"

regulatory 3033..3042

/label=Kozak sequence

/note="vertebrate consensus sequence for strong initiation

of translation (Kozak, 1987)"

/regulatory_class="other"

CDS 3039..3556

/codon_start=1

/product="NanoLuc(R) luciferase"

/label=Nluc

/note="human codon-optimized"

/translation="MVFTLEDFVGDWRQTAGYNLDQVLEQGGVSSLFQNLGVSVTPIQR

IVLSGENGLKIDIHVIIPYEGLSGDQMGQIEKIFKVVYPVDDHHFKVILHYGTLVIDGV

TPNMIDYFGRPYEGIAVFDGKKITVTGTLWNGNKIIDERLINPDGSLLFRVTING*VTG

WRLCERILA"

primer_bind 3447..3465

/label=NanoLueckFseq (121)

misc_feature 3516..3518

/label=PTC

misc_feature 3616..3747

/label=SV40 poly(A) signal

misc_feature 3785..3791

/label=SapI

promoter 3816..3846

/label=lac UV5 promoter

/note="E. coli lac promoter with an ""up"" mutation"

CDS 3900..4559

/codon_start=1

/gene="cat"

/product="chloramphenicol acetyltransferase"

/label=CmR

/note="confers resistance to chloramphenicol"

/translation="MEKKITGYTTVDISQWHRKEHFEAFQSVAQCTYNQTVQLDITAFL

KTVKKNKHKFYPAFIHILARLMNAHPEFRMAMKDGELVIWDSVHPCYTVFHEQTETFSS

LWSEYHDDFRQFLHIYSQDVACYGENLAYFPKGFIENMFFVSANPWVSFTSFDLNVANM

DNFFAPVFTMGKYYTQGDKVLMPLAIQVHHAVCDGFHVGRMLNELQQYCDEWQGGA"

CDS 4901..5206

/codon_start=1

/gene="ccdB"

/product="CcdB, a bacterial toxin that poisons DNA gyrase"

/label=ccdB

/note="Plasmids containing the ccdB gene cannot be

propagated in standard E. coli strains."

/translation="MQFKVYTYKRESRYRLFVDVQSDIIDTPGRRMVIPLASARLLSDK

VSRELYPVVHIGDESWRMMTTDMASVPVSVIGEEVADLSHRENDIKNAINLMFWGI"

misc_feature 5245..5251

/label=SapI

gene complement(5253..5325)

/gene="lacZ"

/label=lacZ

misc_feature 5253..5325

/label=tRNA Arg

variation 5326..5337

/label=3' term signal

/note="3' term signal"

rep_origin complement(5420..6008)

/direction=LEFT

/label=pBR322 ori

/note="high-copy-number ColE1/pMB1/pBR322/pUC origin of

replication"

CDS complement(6179..7039)

/codon_start=1

/gene="bla"

/product="beta-lactamase"

/label=AmpR

/note="confers resistance to ampicillin, carbenicillin, and

related antibiotics"

/translation="MSIQHFRVALIPFFAAFCLPVFAHPETLVKVKDAEDQLGARVGYI

ELDLNSGKILESFRPEERFPMMSTFKVLLCGAVLSRIDAGQEQLGRRIHYSQNDLVEYS

PVTEKHLTDGMTVRELCSAAITMSDNTAANLLLTTIGGPKELTAFLHNMGDHVTRLDRW

EPELNEAIPNDERDTTMPVAMATTLRKLLTGELLTLASRQQLIDWMEADKVAGPLLRSA

LPAGWFIADKSGAGERGSRGIIAALGPDGKPSRIVVIYTTGSQATMDERNRQIAEIGAS

LIKHW"

promoter complement(7040..7144)

/gene="bla"

/label=AmpR promoter

ORIGIN

1 ctgacgtcga cggatcggga gggttaatta cgcgtggcct ccgcgccggg ttttggcgcc

61 tcccgcgggc gcccccctcc tcacggcgag cgctgccacg tcagacgaag ggcgcagcga

121 gcgtcctgat ccttccgccc ggacgctcag gacagcggcc cgctgctcat aagactcggc

181 cttagaaccc cagtatcagc agaaggacat tttaggacgg gacttgggtg actctagggc

241 actggttttc tttccagaga gcggaacagg cgaggaaaag tagtcccttc tcggcgattc

301 tgcggaggga tctccgtggg gcggtgaacg ccgatgatta tataaggacg cgccgggtgt

361 ggcacagcta gttccgtcgc agccgggatt tgggtcgcgg ttcttgtttg tggatcgctg

421 tgatcgtcac ttggttatcg aaattaatac gactcactat agggagaccc aagctgctag

481 ccacgtggcc gccaccatgg aagatgccaa aaacattaag aagggcccag cgccattcta

541 cccactcgag gacgggaccg ccggcgagca gctgcacaaa gccatgaagc gctacgccct

601 ggtgcccggc accatcgcct ttaccgacgc acatatcgag gtggacatta cctacgccga

661 gtacttcgag atgagcgttc ggctggcaga agctatgaag cgctatgggc tgaatacaaa

721 ccatcggatc gtggtgtgca gcgagaatag cttgcagttc ttcatgcccg tgttgggtgc

781 cctgttcatc ggtgtggctg tggccccagc taacgacatc tacaacgagc gcgagctgct

841 gaacagcatg ggcatcagcc agcccaccgt cgtattcgtg agcaagaaag ggctgcaaaa

901 gatcctcaac gtgcaaaaga agctaccgat catacaaaag atcatcatca tggatagcaa

961 gaccgactac cagggcttcc aaagcatgta caccttcgtg acttcccatt tgccacccgg

1021 cttcaacgag tacgacttcg tgcccgagag cttcgaccgg gacaaaacca tcgccctgat

1081 catgaacagt agtggcagta ccggattgcc caagggcgta gccctaccgc accgcaccgc

1141 ttgtgtccga ttcagtcatg cccgcgaccc catcttcggc aaccagatca tccccgacac

1201 cgctatcctc agcgtggtgc catttcacca cggcttcggc atgttcacca cgctgggcta

1261 cttgatctgc ggctttcggg tcgtgctcat gtaccgcttc gaggaggagc tattcttgcg

1321 cagcttgcaa gactataaga ttcaatctgc cctgctggtg cccacactat ttagcttctt

1381 cgctaagagc actctcatcg acaagtacga cctaagcaac ttgcacgaga tcgccagcgg

1441 cggggcgccg ctcagcaagg aggtaggtga ggccgtggcc aaacgcttcc acctaccagg

1501 catccgccag ggctacggcc tgacagaaac aaccagcgcc attctgatca cccccgaagg

1561 ggacgacaag cctggcgcag taggcaaggt ggtgcccttc ttcgaggcta aggtggtgga

1621 cttggacacc ggtaagacac tgggtgtgaa ccagcgcggc gagctgtgcg tccgtggccc

1681 catgatcatg agcggctacg ttaacaaccc cgaggctaca aacgctctca tcgacaagga

1741 cggctggctg cacagcggcg acatcgccta ctgggacgag gacgagcact tcttcatcgt

1801 ggaccggctg aaaagcctga tcaaatacaa gggctaccag gtagccccag ccgaactgga

1861 gagcatcctg ctgcaacacc ccaacatctt cgacgccggg gtcgccggcc tgcccgacga

1921 cgatgccggc gagctgcccg ccgcagtcgt cgtgctggaa cacggtaaaa ccatgaccga

1981 gaaggagatc gtggactatg tggccagcca ggttacaacc gccaagaagc tgcgcggtgg

2041 tgttgtgttc gtggacgagg tgcctaaagg actgaccggc aagttggacg cccgcaagat

2101 ccgcgagatt ctcattaagg ccaagaaggg cggcaagatc gccgtgtaat gatagggtac

2161 cttgtttatt gcagcttata atggttacaa ataaagcaat agcatcacaa atttcacaaa

2221 taaagcattt ttttcactgc attctagttg tggtttgtcc aaactcatca atgtatctta

2281 tgcatgaaga atctgcttag ggttaggcgt tttgcgctgc ttcgcgatgt acgggccaga

2341 tatacgcgtt gacattgatt attgactagt tattaatagt aatcaattac ggggtcatta

2401 gttcatagcc catatatgga gttccgcgtt acataactta cggtaaatgg cccgcctggc

2461 tgaccgccca acgacccccg cccattgacg tcaataatga cgtatgttcc catagtaacg

2521 ccaataggga ctttccattg acgtcaatgg gtggagtatt tacggtaaac tgcccacttg

2581 gcagtacatc aagtgtatca tatgccaagt acgcccccta ttgacgtcaa tgacggtaaa

2641 tggcccgcct ggcattatgc ccagtacatg accttatggg actttcctac ttggcagtac

2701 atctacgtat tagtcatcgc tattaccatg gtgatgcggt tttggcagta catcaatggg

2761 cgtggatagc ggtttgactc acggggattt ccaagtctcc accccattga cgtcaatggg

2821 agtttgtttt ggcaccaaaa tcaacgggac tttccaaaat gtcgtaacaa ctccgcccca

2881 ttgacgcaaa tgggcggtag gcgtgtacgg tgggaggtct atataagcag agctctctgg

2941 ctaactagag aacccactgc ttactggctt atcgaaatta atacgactca ctatagggag

3001 acccaagctg gctagcgttt aaacttaagc ttgccaccat ggtattcaca ctcgaagatt

3061 tcgttgggga ctggcgacag acagccggct acaacctgga ccaagtcctt gaacagggag

3121 gtgtgtccag tttgtttcag aatctcgggg tgtccgtaac tccgatccaa aggattgtcc

3181 tgagcggtga aaatgggctg aagatcgaca tccatgtcat catcccgtat gaaggtctga

3241 gcggcgacca aatgggccag atcgaaaaaa tttttaaggt ggtgtaccct gtggatgatc

3301 atcactttaa ggtgatcctg cactatggca cactggtaat cgacggggtt acgccgaaca

3361 tgatcgacta tttcggacgg ccgtatgaag gcatcgccgt gttcgacggc aaaaagatca

3421 ctgtaacagg gaccctgtgg aacggcaaca aaattatcga cgagcgcctg atcaaccccg

3481 acggctccct gctgttccga gtaaccatca acggatgagt gaccggctgg cggctgtgcg

3541 aacgcattct ggcgtaagaa ttctaagggc gaattctgca gatatccagc acagtggcgg

3601 ccgctcgagt ctagattgtt tattgcagct tataatggtt acaaataaag caatagcatc

3661 acaaatttca caaataaagc atttttttca ctgcattcta gttgtggttt gtccaaactc

3721 atcaatgtat cttatcatgt ctggatcgct agagggcctt cctaatacga ctcactatag

3781 tttcgaagag cgcggccgca ttaggcaccc caggctttac actttatgct tccggctcgt

3841 ataatgtgtg gattttgagt taggatccgt cgagattttc aggagctaag gaagctaaaa

3901 tggagaaaaa aatcactgga tataccaccg ttgatatatc ccaatggcat cgtaaagaac

3961 attttgaggc atttcagtca gttgctcaat gtacctataa ccagaccgtt cagctggata

4021 ttacggcctt tttaaagacc gtaaagaaaa ataagcacaa gttttatccg gcctttattc

4081 acattcttgc ccgcctgatg aatgctcatc cggaattccg tatggcaatg aaagacggtg

4141 agctggtgat atgggatagt gttcaccctt gttacaccgt tttccatgag caaactgaaa

4201 cgttttcatc gctctggagt gaataccacg acgatttccg gcagtttcta cacatatatt

4261 cgcaagatgt ggcgtgttac ggtgaaaacc tggcctattt ccctaaaggg tttattgaga

4321 atatgttttt cgtctcagcc aatccctggg tgagtttcac cagttttgat ttaaacgtgg

4381 ccaatatgga caacttcttc gcccccgttt tcaccatggg caaatattat acgcaaggcg

4441 acaaggtgct gatgccgctg gcgattcagg ttcatcatgc cgtttgtgat ggcttccatg

4501 tcggcagaat gcttaatgaa ttacaacagt actgcgatga gtggcagggc ggggcgtaaa

4561 gatctggatc cggcttacta aaagccagat aacagtatgc gtatttgcgc gctgattttt

4621 gcggtataag aatatatact gatatgtata cccgaagtat gtcaaaaaga ggtatgctat

4681 gaagcagcgt attacagtga cagttgacag cgacagctat cagttgctca aggcatatat

4741 gatgtcaata tctccggtct ggtaagcaca accatgcaga atgaagcccg tcgtctgcgt

4801 gccgaacgct ggaaagcgga aaatcaggaa gggatggctg aggtcgcccg gtttattgaa

4861 atgaacggct cttttgctga cgagaacagg ggctggtgaa atgcagttta aggtttacac

4921 ctataaaaga gagagccgtt atcgtctgtt tgtggatgta cagagtgata ttattgacac

4981 gcccgggcga cggatggtga tccccctggc cagtgcacgt ctgctgtcag ataaagtctc

5041 ccgtgaactt tacccggtgg tgcatatcgg ggatgaaagc tggcgcatga tgaccaccga

5101 tatggccagt gtgcccgtct ccgttatcgg ggaagaagtg gctgatctca gccaccgcga

5161 aaatgacatc aaaaacgcca ttaacctgat gttctgggga atataaatgt caggctccct

5221 tatacacagc cagtctgcag ggaagctctt cgggctctgt ggcgcaatgg atagcgcatt

5281 ggacttcaaa ttcaaaggtt gtgggttcga gtcccaccag agtcggtcct ttttttgctt

5341 tagtgagggt taattcaggc atgtgagcaa aaggccagca aaaggccagg aaccgtaaaa

5401 aggccgcgtt gctggcgttt ttccataggc tccgcccccc tgacgagcat cacaaaaatc

5461 gacgctcaag tcagaggtgg cgaaacccga caggactata aagataccag gcgtttcccc

5521 ctggaagctc cctcgtgcgc tctcctgttc cgaccctgcc gcttaccgga tacctgtccg

5581 cctttctccc ttcgggaagc gtggcgcttt ctcaatgctc acgctgtagg tatctcagtt

5641 cggtgtaggt cgttcgctcc aagctgggct gtgtgcacga accccccgtt cagcccgacc

5701 gctgcgcctt atccggtaac tatcgtcttg agtccaaccc ggtaagacac gacttatcgc

5761 cactggcagc agccactggt aacaggatta gcagagcgag gtatgtaggc ggtgctacag

5821 agttcttgaa gtggtggcct aactacggct acactagaag gacagtattt ggtatctgcg

5881 ctctgctgaa gccagttacc ttcggaaaaa gagttggtag ctcttgatcc ggcaaacaaa

5941 ccaccgctac cagcggtggt ttttttgttt gcaagcagca gattacgcgc agaaaaaaag

6001 gatctcaaga agatcctttg atcttttcta cggggtctga cgctcagtgg aacgaaaact

6061 cacgttaagg gattttggtc atgagattat caaaaaggat cttcacctag atccttttaa

6121 attaaaaatg aagttttaaa tcaatctaaa gtatatatga gtaaacttgg tctgacagtt

6181 accaatgctt aatcagtgag gcacctatct cagcgatctg tctatttcgt tcatccatag

6241 ttgcctgact ccccgtcgtg tagataacta cgatacggga gggcttacca tctggcccca

6301 gtgctgcaat gataccgcga gacccacgct caccggctcc agatttatca gcaataaacc

6361 agccagccgg aagggccgag cgcagaagtg gtcctgcaac tttatccgcc tccatccagt

6421 ctattaattg ttgccgggaa gctagagtaa gtagttcgcc agttaatagt ttgcgcaacg

6481 ttgttgccat tgctacaggc atcgtggtgt cacgctcgtc gtttggtatg gcttcattca

6541 gctccggttc ccaacgatca aggcgagtta catgatcccc catgttgtgc aaaaaagcgg

6601 ttagctcctt cggtcctccg atcgttgtca gaagtaagtt ggccgcagtg ttatcactca

6661 tggttatggc agcactgcat aattctctta ctgtcatgcc atccgtaaga tgcttttctg

6721 tgactggtga gtactcaacc aagtcattct gagaatagtg tatgcggcga ccgagttgct

6781 cttgcccggc gtcaatacgg gataataccg cgccacatag cagaacttta aaagtgctca

6841 tcattggaaa acgttcttcg gggcgaaaac tctcaaggat cttaccgctg ttgagatcca

6901 gttcgatgta acccactcgt gcacccaact gatcttcagc atcttttact ttcaccagcg

6961 tttctgggtg agcaaaaaca ggaaggcaaa atgccgcaaa aaagggaata agggcgacac

7021 ggaaatgttg aatactcata ctcttccttt ttcaatatta ttgaagcatt tatcagggtt

7081 attgtctcat gagcggatac atatttgaat gtatttagaa aaataaacaa ataggggttc

7141 cgcgcacatt tccccgaaaa gtgccac

//

**Genbank sequence for 3’ HTCS vector**

LOCUS 3prime_pNanoRePo 7211 bp DNA circular SYN 29-MAR-2023

DEFINITION synthetic circular DNA

ACCESSION .

VERSION .

KEYWORDS .

SOURCE synthetic DNA construct

ORGANISM synthetic DNA construct

REFERENCE 1 (bases 1 to 7211)

AUTHORS .

TITLE Direct Submission

JOURNAL Exported Mar 29, 2023 from SnapGene 6.2.1

https://www.snapgene.com

COMMENT LOCUS pcDNA3.1/Zeo(+) 5015 bp DNA SYN

DEFINITION pcDNA3.1/Zeo(+)

ACCESSION

KEYWORDS

SOURCE

ORGANISM other sequences; artificial sequences; vectors.

FEATURES Location/Qualifiers

source 1..7211

/mol_type="other DNA"

/organism="synthetic DNA construct"

source 3836..3908

/lab_host="E.coli"

/db_xref="taxon:66198"

/organism="Cloning vector pUC57"

promoter 36..435

/label=UbC promoter

/note="human ubiquitin C promoter"

promoter 445..463

/label=T7 promoter

/note="promoter for bacteriophage T7 RNA polymerase"

regulatory 491..500

/label=Kozak sequence

/note="vertebrate consensus sequence for strong initiation

of translation (Kozak, 1987)"

/regulatory_class="other"

CDS 497..2146

/codon_start=1

/product="firefly luciferase reporter gene luc2 (Photinus

pyralis) and is designed for high expression and reduced

anomalous transcription. This sequence was engineered with

fewer consensus regulatory sequences and has been codon

optimized for mammalian expression."

/label=Fluc2 (From Promega pGL4.10[luc2] vector)

/note="synthetic luc2 version of the luciferase gene"

/translation="MEDAKNIKKGPAPFYPLEDGTAGEQLHKAMKRYALVPGTIAFTDA

HIEVDITYAEYFEMSVRLAEAMKRYGLNTNHRIVVCSENSLQFFMPVLGALFIGVAVAP

ANDIYNERELLNSMGISQPTVVFVSKKGLQKILNVQKKLPIIQKIIIMDSKTDYQGFQS

MYTFVTSHLPPGFNEYDFVPESFDRDKTIALIMNSSGSTGLPKGVALPHRTACVRFSHA

RDPIFGNQIIPDTAILSVVPFHHGFGMFTTLGYLICGFRVVLMYRFEEELFLRSLQDYK

IQSALLVPTLFSFFAKSTLIDKYDLSNLHEIASGGAPLSKEVGEAVAKRFHLPGIRQGY

GLTETTSAILITPEGDDKPGAVGKVVPFFEAKVVDLDTGKTLGVNQRGELCVRGPMIMS

GYVNNPEATNALIDKDGWLHSGDIAYWDEDEHFFIVDRLKSLIKYKGYQVAPAELESIL

LQHPNIFDAGVAGLPDDDAGELPAAVVVLEHGKTMTEKEIVDYVASQVTTAKKLRGGVV

FVDEVPKGLTGKLDARKIREILIKAKKGGKIAV"

misc_feature 2162..2281

/label=SV40 poly(A) signal

enhancer 2351..2730

/label=CMV immediate early enhancer

/note="human cytomegalovirus immediate early enhancer"

promoter 2731..2934

/label=CMV promoter

/note="human cytomegalovirus (CMV) immediate early

promoter"

promoter 2979..2997

/label=T7 promoter

/note="promoter for bacteriophage T7 RNA polymerase"

regulatory 3033..3042

/label=Kozak sequence

/note="vertebrate consensus sequence for strong initiation

of translation (Kozak, 1987)"

/regulatory_class="other"

CDS 3039..3556

/codon_start=1

/product="NanoLuc(R) luciferase"

/label=Nluc

/note="human codon-optimized"

/translation="MVFTLEDFVGDWRQTAGYNLDQVLEQGGVSSLFQNLGVSVTPIQR

IVLSGENGLKIDIHVIIPYEGLSGDQMGQIEKIFKVVYPVDDHHFKVILHYGTLVIDGV

TPNMIDYFGRPYEGIAVFDGKKITVTGTLWNGNKIIDERLINPDGSLLFRVTING*VTG

WRLCERILA"

primer_bind 3447..3465

/label=NanoLueckFseq (121)

misc_feature 3516..3518

/label=PTC

misc_feature 3616..3747

/label=SV40 poly(A) signal

5'UTR 3763..3835

/label=5' human Tyr-tRNA leader

/note="5' human Tyr-tRNA leader"

gene complement(3836..3908)

/gene="lacZ"

/label=lacZ

misc_feature 3836..3908

/label=tRNA Arg

misc_feature 3911..3916

/label=BbsI

promoter 3941..3971

/label=lac UV5 promoter

/note="E. coli lac promoter with an ""up"" mutation"

CDS 4025..4684

/codon_start=1

/gene="cat"

/product="chloramphenicol acetyltransferase"

/label=CmR

/note="confers resistance to chloramphenicol"

/translation="MEKKITGYTTVDISQWHRKEHFEAFQSVAQCTYNQTVQLDITAFL

KTVKKNKHKFYPAFIHILARLMNAHPEFRMAMKDGELVIWDSVHPCYTVFHEQTETFSS

LWSEYHDDFRQFLHIYSQDVACYGENLAYFPKGFIENMFFVSANPWVSFTSFDLNVANM

DNFFAPVFTMGKYYTQGDKVLMPLAIQVHHAVCDGFHVGRMLNELQQYCDEWQGGA"

CDS 5026..5331

/codon_start=1

/gene="ccdB"

/product="CcdB, a bacterial toxin that poisons DNA gyrase"

/label=ccdB

/note="Plasmids containing the ccdB gene cannot be

propagated in standard E. coli strains."

/translation="MQFKVYTYKRESRYRLFVDVQSDIIDTPGRRMVIPLASARLLSDK

VSRELYPVVHIGDESWRMMTTDMASVPVSVIGEEVADLSHRENDIKNAINLMFWGI"

misc_feature 5370..5375

/label=BbsI

rep_origin complement(5464..6052)

/direction=LEFT

/label=pBR322 ori

/note="high-copy-number ColE1/pMB1/pBR322/pUC origin of

replication"

CDS complement(6223..7083)

/codon_start=1

/gene="bla"

/product="beta-lactamase"

/label=AmpR

/note="confers resistance to ampicillin, carbenicillin, and

related antibiotics"

/translation="MSIQHFRVALIPFFAAFCLPVFAHPETLVKVKDAEDQLGARVGYI

ELDLNSGKILESFRPEERFPMMSTFKVLLCGAVLSRIDAGQEQLGRRIHYSQNDLVEYS

PVTEKHLTDGMTVRELCSAAITMSDNTAANLLLTTIGGPKELTAFLHNMGDHVTRLDRW

EPELNEAIPNDERDTTMPVAMATTLRKLLTGELLTLASRQQLIDWMEADKVAGPLLRSA

LPAGWFIADKSGAGERGSRGIIAALGPDGKPSRIVVIYTTGSQATMDERNRQIAEIGAS

LIKHW"

promoter complement(7084..7188)

/gene="bla"

/label=AmpR promoter

ORIGIN

1 ctgacgtcga cggatcggga gggttaatta cgcgtggcct ccgcgccggg ttttggcgcc

61 tcccgcgggc gcccccctcc tcacggcgag cgctgccacg tcagacgaag ggcgcagcga

121 gcgtcctgat ccttccgccc ggacgctcag gacagcggcc cgctgctcat aagactcggc

181 cttagaaccc cagtatcagc agaaggacat tttaggacgg gacttgggtg actctagggc

241 actggttttc tttccagaga gcggaacagg cgaggaaaag tagtcccttc tcggcgattc

301 tgcggaggga tctccgtggg gcggtgaacg ccgatgatta tataaggacg cgccgggtgt

361 ggcacagcta gttccgtcgc agccgggatt tgggtcgcgg ttcttgtttg tggatcgctg

421 tgatcgtcac ttggttatcg aaattaatac gactcactat agggagaccc aagctgctag

481 ccacgtggcc gccaccatgg aagatgccaa aaacattaag aagggcccag cgccattcta

541 cccactcgag gacgggaccg ccggcgagca gctgcacaaa gccatgaagc gctacgccct

601 ggtgcccggc accatcgcct ttaccgacgc acatatcgag gtggacatta cctacgccga

661 gtacttcgag atgagcgttc ggctggcaga agctatgaag cgctatgggc tgaatacaaa

721 ccatcggatc gtggtgtgca gcgagaatag cttgcagttc ttcatgcccg tgttgggtgc

781 cctgttcatc ggtgtggctg tggccccagc taacgacatc tacaacgagc gcgagctgct

841 gaacagcatg ggcatcagcc agcccaccgt cgtattcgtg agcaagaaag ggctgcaaaa

901 gatcctcaac gtgcaaaaga agctaccgat catacaaaag atcatcatca tggatagcaa

961 gaccgactac cagggcttcc aaagcatgta caccttcgtg acttcccatt tgccacccgg

1021 cttcaacgag tacgacttcg tgcccgagag cttcgaccgg gacaaaacca tcgccctgat

1081 catgaacagt agtggcagta ccggattgcc caagggcgta gccctaccgc accgcaccgc

1141 ttgtgtccga ttcagtcatg cccgcgaccc catcttcggc aaccagatca tccccgacac

1201 cgctatcctc agcgtggtgc catttcacca cggcttcggc atgttcacca cgctgggcta

1261 cttgatctgc ggctttcggg tcgtgctcat gtaccgcttc gaggaggagc tattcttgcg

1321 cagcttgcaa gactataaga ttcaatctgc cctgctggtg cccacactat ttagcttctt

1381 cgctaagagc actctcatcg acaagtacga cctaagcaac ttgcacgaga tcgccagcgg

1441 cggggcgccg ctcagcaagg aggtaggtga ggccgtggcc aaacgcttcc acctaccagg

1501 catccgccag ggctacggcc tgacagaaac aaccagcgcc attctgatca cccccgaagg

1561 ggacgacaag cctggcgcag taggcaaggt ggtgcccttc ttcgaggcta aggtggtgga

1621 cttggacacc ggtaagacac tgggtgtgaa ccagcgcggc gagctgtgcg tccgtggccc

1681 catgatcatg agcggctacg ttaacaaccc cgaggctaca aacgctctca tcgacaagga

1741 cggctggctg cacagcggcg acatcgccta ctgggacgag gacgagcact tcttcatcgt

1801 ggaccggctg aagagcctga tcaaatacaa gggctaccag gtagccccag ccgaactgga

1861 gagcatcctg ctgcaacacc ccaacatctt cgacgccggg gtcgccggcc tgcccgacga

1921 cgatgccggc gagctgcccg ccgcagtcgt cgtgctggaa cacggtaaaa ccatgaccga

1981 gaaggagatc gtggactatg tggccagcca ggttacaacc gccaagaagc tgcgcggtgg

2041 tgttgtgttc gtggacgagg tgcctaaagg actgaccggc aagttggacg cccgcaagat

2101 ccgcgagatt ctcattaagg ccaagaaggg cggcaagatc gccgtgtaat gatagggtac

2161 cttgtttatt gcagcttata atggttacaa ataaagcaat agcatcacaa atttcacaaa

2221 taaagcattt ttttcactgc attctagttg tggtttgtcc aaactcatca atgtatctta

2281 tgcatgaaga atctgcttag ggttaggcgt tttgcgctgc ttcgcgatgt acgggccaga

2341 tatacgcgtt gacattgatt attgactagt tattaatagt aatcaattac ggggtcatta

2401 gttcatagcc catatatgga gttccgcgtt acataactta cggtaaatgg cccgcctggc

2461 tgaccgccca acgacccccg cccattgacg tcaataatga cgtatgttcc catagtaacg

2521 ccaataggga ctttccattg acgtcaatgg gtggagtatt tacggtaaac tgcccacttg

2581 gcagtacatc aagtgtatca tatgccaagt acgcccccta ttgacgtcaa tgacggtaaa

2641 tggcccgcct ggcattatgc ccagtacatg accttatggg actttcctac ttggcagtac

2701 atctacgtat tagtcatcgc tattaccatg gtgatgcggt tttggcagta catcaatggg

2761 cgtggatagc ggtttgactc acggggattt ccaagtctcc accccattga cgtcaatggg

2821 agtttgtttt ggcaccaaaa tcaacgggac tttccaaaat gtcgtaacaa ctccgcccca

2881 ttgacgcaaa tgggcggtag gcgtgtacgg tgggaggtct atataagcag agctctctgg

2941 ctaactagag aacccactgc ttactggctt atcgaaatta atacgactca ctatagggag

3001 acccaagctg gctagcgttt aaacttaagc ttgccaccat ggtattcaca ctcgaagatt

3061 tcgttgggga ctggcgacag acagccggct acaacctgga ccaagtcctt gaacagggag

3121 gtgtgtccag tttgtttcag aatctcgggg tgtccgtaac tccgatccaa aggattgtcc

3181 tgagcggtga aaatgggctg aagatcgaca tccatgtcat catcccgtat gaaggtctga

3241 gcggcgacca aatgggccag atcgaaaaaa tttttaaggt ggtgtaccct gtggatgatc

3301 atcactttaa ggtgatcctg cactatggca cactggtaat cgacggggtt acgccgaaca

3361 tgatcgacta tttcggacgg ccgtatgaag gcatcgccgt gttcgacggc aaaaagatca

3421 ctgtaacagg gaccctgtgg aacggcaaca aaattatcga cgagcgcctg atcaaccccg

3481 acggctccct gctgttccga gtaaccatca acggatgagt gaccggctgg cggctgtgcg

3541 aacgcattct ggcgtaagaa ttctaagggc gaattctgca gatatccagc acagtggcgg

3601 ccgctcgagt ctagattgtt tattgcagct tataatggtt acaaataaag caatagcatc

3661 acaaatttca caaataaagc atttttttca ctgcattcta gttgtggttt gtccaaactc

3721 atcaatgtat cttatcatgt ctggatcgct agagggcctt cctaatacga ctcactatag

3781 agcgctccgg tttttctgtg ctgaacctca ggggacgccg acacacgtac acgtcggctc

3841 tgtggcgcaa tggatagcgc attggacttc aaattcaaag gttgtgggtt cgagtcccac

3901 cagagtcgta gtcttcgcgg ccgcattagg caccccaggc tttacacttt atgcttccgg

3961 ctcgtataat gtgtggattt tgagttagga tccgtcgaga ttttcaggag ctaaggaagc

4021 taaaatggag aaaaaaatca ctggatatac caccgttgat atatcccaat ggcatcgtaa

4081 agaacatttt gaggcatttc agtcagttgc tcaatgtacc tataaccaga ccgttcagct

4141 ggatattacg gcctttttaa agaccgtaaa gaaaaataag cacaagtttt atccggcctt

4201 tattcacatt cttgcccgcc tgatgaatgc tcatccggaa ttccgtatgg caatgaaaga

4261 cggtgagctg gtgatatggg atagtgttca cccttgttac accgttttcc atgagcaaac

4321 tgaaacgttt tcatcgctct ggagtgaata ccacgacgat ttccggcagt ttctacacat

4381 atattcgcaa gatgtggcgt gttacggtga aaacctggcc tatttcccta aagggtttat

4441 tgagaatatg tttttcgtct cagccaatcc ctgggtgagt ttcaccagtt ttgatttaaa

4501 cgtggccaat atggacaact tcttcgcccc cgttttcacc atgggcaaat attatacgca

4561 aggcgacaag gtgctgatgc cgctggcgat tcaggttcat catgccgttt gtgatggctt

4621 ccatgtcggc agaatgctta atgaattaca acagtactgc gatgagtggc agggcggggc

4681 gtaaagatct ggatccggct tactaaaagc cagataacag tatgcgtatt tgcgcgctga

4741 tttttgcggt ataagaatat atactgatat gtatacccga agtatgtcaa aaagaggtat

4801 gctatgaagc agcgtattac agtgacagtt gacagcgaca gctatcagtt gctcaaggca

4861 tatatgatgt caatatctcc ggtctggtaa gcacaaccat gcagaatgaa gcccgtcgtc

4921 tgcgtgccga acgctggaaa gcggaaaatc aggaagggat ggctgaggtc gcccggttta

4981 ttgaaatgaa cggctctttt gctgacgaga acaggggctg gtgaaatgca gtttaaggtt

5041 tacacctata aaagagagag ccgttatcgt ctgtttgtgg atgtacagag tgatattatt

5101 gacacgcccg ggcgacggat ggtgatcccc ctggccagtg cacgtctgct gtcagataaa

5161 gtctcccgtg aactttaccc ggtggtgcat atcggggatg aaagctggcg catgatgacc

5221 accgatatgg ccagtgtgcc cgtctccgtt atcggggaag aagtggctga tctcagccac

5281 cgcgaaaatg acatcaaaaa cgccattaac ctgatgttct ggggaatata aatgtcaggc

5341 tcccttatac acagccagtc tgcagggaag aagaccggtc cctttagtga gggttaattc

5401 aggcatgtga gcaaaaggcc agcaaaaggc caggaaccgt aaaaaggccg cgttgctggc

5461 gtttttccat aggctccgcc cccctgacga gcatcacaaa aatcgacgct caagtcagag

5521 gtggcgaaac ccgacaggac tataaagata ccaggcgttt ccccctggaa gctccctcgt

5581 gcgctctcct gttccgaccc tgccgcttac cggatacctg tccgcctttc tcccttcggg

5641 aagcgtggcg ctttctcaat gctcacgctg taggtatctc agttcggtgt aggtcgttcg

5701 ctccaagctg ggctgtgtgc acgaaccccc cgttcagccc gaccgctgcg ccttatccgg

5761 taactatcgt cttgagtcca acccggtaag acacgactta tcgccactgg cagcagccac

5821 tggtaacagg attagcagag cgaggtatgt aggcggtgct acagagttct tgaagtggtg

5881 gcctaactac ggctacacta gaaggacagt atttggtatc tgcgctctgc tgaagccagt

5941 taccttcgga aaaagagttg gtagctcttg atccggcaaa caaaccaccg ctaccagcgg

6001 tggttttttt gtttgcaagc agcagattac gcgcagaaaa aaaggatctc aagaagatcc

6061 tttgatcttt tctacggggt ctgacgctca gtggaacgaa aactcacgtt aagggatttt

6121 ggtcatgaga ttatcaaaaa ggatcttcac ctagatcctt ttaaattaaa aatgaagttt

6181 taaatcaatc taaagtatat atgagtaaac ttggtctgac agttaccaat gcttaatcag

6241 tgaggcacct atctcagcga tctgtctatt tcgttcatcc atagttgcct gactccccgt

6301 cgtgtagata actacgatac gggagggctt accatctggc cccagtgctg caatgatacc

6361 gcgagaccca cgctcaccgg ctccagattt atcagcaata aaccagccag ccggaagggc

6421 cgagcgcaga agtggtcctg caactttatc cgcctccatc cagtctatta attgttgccg

6481 ggaagctaga gtaagtagtt cgccagttaa tagtttgcgc aacgttgttg ccattgctac

6541 aggcatcgtg gtgtcacgct cgtcgtttgg tatggcttca ttcagctccg gttcccaacg

6601 atcaaggcga gttacatgat cccccatgtt gtgcaaaaaa gcggttagct ccttcggtcc

6661 tccgatcgtt gtcagaagta agttggccgc agtgttatca ctcatggtta tggcagcact

6721 gcataattct cttactgtca tgccatccgt aagatgcttt tctgtgactg gtgagtactc

6781 aaccaagtca ttctgagaat agtgtatgcg gcgaccgagt tgctcttgcc cggcgtcaat

6841 acgggataat accgcgccac atagcagaac tttaaaagtg ctcatcattg gaaaacgttc

6901 ttcggggcga aaactctcaa ggatcttacc gctgttgaga tccagttcga tgtaacccac

6961 tcgtgcaccc aactgatctt cagcatcttt tactttcacc agcgtttctg ggtgagcaaa

7021 aacaggaagg caaaatgccg caaaaaaggg aataagggcg acacggaaat gttgaatact

7081 catactcttc ctttttcaat attattgaag catttatcag ggttattgtc tcatgagcgg

7141 atacatattt gaatgtattt agaaaaataa acaaataggg gttccgcgca catttccccg

7201 aaaagtgcca c

//

**Genbank sequence for tRNA HTCS vector**

LOCUS tRNA_screening_p 7146 bp DNA circular SYN 29-MAR-2023

DEFINITION synthetic circular DNA

ACCESSION .

VERSION .

KEYWORDS .

SOURCE synthetic DNA construct

ORGANISM synthetic DNA construct

REFERENCE 1 (bases 1 to 7146)

AUTHORS .

TITLE Direct Submission

JOURNAL Exported Mar 29, 2023 from SnapGene 6.2.1

https://www.snapgene.com

COMMENT LOCUS pcDNA3.1/Zeo(+) 5015 bp DNA SYN

DEFINITION pcDNA3.1/Zeo(+)

ACCESSION

KEYWORDS

SOURCE

ORGANISM other sequences; artificial sequences; vectors.

FEATURES Location/Qualifiers

source 1..7146

/mol_type="other DNA"

/organism="synthetic DNA construct"

promoter 36..435

/label=UbC promoter

/note="human ubiquitin C promoter"

promoter 445..463

/label=T7 promoter

/note="promoter for bacteriophage T7 RNA polymerase"

regulatory 491..500

/label=Kozak sequence

/note="vertebrate consensus sequence for strong initiation

of translation (Kozak, 1987)"

/regulatory_class="other"

CDS 497..2146

/codon_start=1

/product="firefly luciferase reporter gene luc2 (Photinus

pyralis) and is designed for high expression and reduced

anomalous transcription. This sequence was engineered with

fewer consensus regulatory sequences and has been codon

optimized for mammalian expression."

/label=Fluc2 (From Promega pGL4.10[luc2] vector)

/note="synthetic luc2 version of the luciferase gene"

/translation="MEDAKNIKKGPAPFYPLEDGTAGEQLHKAMKRYALVPGTIAFTDA

HIEVDITYAEYFEMSVRLAEAMKRYGLNTNHRIVVCSENSLQFFMPVLGALFIGVAVAP

ANDIYNERELLNSMGISQPTVVFVSKKGLQKILNVQKKLPIIQKIIIMDSKTDYQGFQS

MYTFVTSHLPPGFNEYDFVPESFDRDKTIALIMNSSGSTGLPKGVALPHRTACVRFSHA

RDPIFGNQIIPDTAILSVVPFHHGFGMFTTLGYLICGFRVVLMYRFEEELFLRSLQDYK

IQSALLVPTLFSFFAKSTLIDKYDLSNLHEIASGGAPLSKEVGEAVAKRFHLPGIRQGY

GLTETTSAILITPEGDDKPGAVGKVVPFFEAKVVDLDTGKTLGVNQRGELCVRGPMIMS

GYVNNPEATNALIDKDGWLHSGDIAYWDEDEHFFIVDRLKSLIKYKGYQVAPAELESIL

LQHPNIFDAGVAGLPDDDAGELPAAVVVLEHGKTMTEKEIVDYVASQVTTAKKLRGGVV

FVDEVPKGLTGKLDARKIREILIKAKKGGKIAV"

misc_feature 2162..2281

/label=SV40 poly(A) signal

enhancer 2351..2730

/label=CMV immediate early enhancer

/note="human cytomegalovirus immediate early enhancer"

promoter 2731..2934

/label=CMV promoter

/note="human cytomegalovirus (CMV) immediate early

promoter"

promoter 2979..2997

/label=T7 promoter

/note="promoter for bacteriophage T7 RNA polymerase"

regulatory 3033..3042

/label=Kozak sequence

/note="vertebrate consensus sequence for strong initiation

of translation (Kozak, 1987)"

/regulatory_class="other"

CDS 3039..3556

/codon_start=1

/product="NanoLuc(R) luciferase"

/label=Nluc

/note="human codon-optimized"

/translation="MVFTLEDFVGDWRQTAGYNLDQVLEQGGVSSLFQNLGVSVTPIQR

IVLSGENGLKIDIHVIIPYEGLSGDQMGQIEKIFKVVYPVDDHHFKVILHYGTLVIDGV

TPNMIDYFGRPYEGIAVFDGKKITVTGTLWNGNKIIDERLINPDGSLLFRVTING*VTG

WRLCERILA"

primer_bind 3447..3465

/label=NanoLueckFseq (121)

misc_feature 3516..3518

/label=PTC

misc_feature 3616..3747

/label=SV40 poly(A) signal

5'UTR 3763..3835

/label=5' human Tyr-tRNA leader

/note="5' human Tyr-tRNA leader"

misc_feature 3838..3843

/label=BbsI

promoter 3868..3898

/label=lac UV5 promoter

/note="E. coli lac promoter with an ""up"" mutation"

CDS 3952..4611

/codon_start=1

/gene="cat"

/product="chloramphenicol acetyltransferase"

/label=CmR

/note="confers resistance to chloramphenicol"

/translation="MEKKITGYTTVDISQWHRKEHFEAFQSVAQCTYNQTVQLDITAFL

KTVKKNKHKFYPAFIHILARLMNAHPEFRMAMKDGELVIWDSVHPCYTVFHEQTETFSS

LWSEYHDDFRQFLHIYSQDVACYGENLAYFPKGFIENMFFVSANPWVSFTSFDLNVANM

DNFFAPVFTMGKYYTQGDKVLMPLAIQVHHAVCDGFHVGRMLNELQQYCDEWQGGA"

CDS 4953..5258

/codon_start=1

/gene="ccdB"

/product="CcdB, a bacterial toxin that poisons DNA gyrase"

/label=ccdB

/note="Plasmids containing the ccdB gene cannot be

propagated in standard E. coli strains."

/translation="MQFKVYTYKRESRYRLFVDVQSDIIDTPGRRMVIPLASARLLSDK

VSRELYPVVHIGDESWRMMTTDMASVPVSVIGEEVADLSHRENDIKNAINLMFWGI"

misc_feature 5297..5302

/label=BbsI

variation 5305..5316

/label=3' term signal

/note="3' term signal"

rep_origin complement(5399..5987)

/direction=LEFT

/label=pBR322 ori

/note="high-copy-number ColE1/pMB1/pBR322/pUC origin of

replication"

CDS complement(6158..7018)

/codon_start=1

/gene="bla"

/product="beta-lactamase"

/label=AmpR

/note="confers resistance to ampicillin, carbenicillin, and

related antibiotics"

/translation="MSIQHFRVALIPFFAAFCLPVFAHPETLVKVKDAEDQLGARVGYI

ELDLNSGKILESFRPEERFPMMSTFKVLLCGAVLSRIDAGQEQLGRRIHYSQNDLVEYS

PVTEKHLTDGMTVRELCSAAITMSDNTAANLLLTTIGGPKELTAFLHNMGDHVTRLDRW

EPELNEAIPNDERDTTMPVAMATTLRKLLTGELLTLASRQQLIDWMEADKVAGPLLRSA

LPAGWFIADKSGAGERGSRGIIAALGPDGKPSRIVVIYTTGSQATMDERNRQIAEIGAS

LIKHW"

promoter complement(7019..7123)

/gene="bla"

/label=AmpR promoter

ORIGIN

1 ctgacgtcga cggatcggga gggttaatta cgcgtggcct ccgcgccggg ttttggcgcc

61 tcccgcgggc gcccccctcc tcacggcgag cgctgccacg tcagacgaag ggcgcagcga

121 gcgtcctgat ccttccgccc ggacgctcag gacagcggcc cgctgctcat aagactcggc

181 cttagaaccc cagtatcagc agaaggacat tttaggacgg gacttgggtg actctagggc

241 actggttttc tttccagaga gcggaacagg cgaggaaaag tagtcccttc tcggcgattc

301 tgcggaggga tctccgtggg gcggtgaacg ccgatgatta tataaggacg cgccgggtgt

361 ggcacagcta gttccgtcgc agccgggatt tgggtcgcgg ttcttgtttg tggatcgctg

421 tgatcgtcac ttggttatcg aaattaatac gactcactat agggagaccc aagctgctag

481 ccacgtggcc gccaccatgg aagatgccaa aaacattaag aagggcccag cgccattcta

541 cccactcgag gacgggaccg ccggcgagca gctgcacaaa gccatgaagc gctacgccct

601 ggtgcccggc accatcgcct ttaccgacgc acatatcgag gtggacatta cctacgccga

661 gtacttcgag atgagcgttc ggctggcaga agctatgaag cgctatgggc tgaatacaaa

721 ccatcggatc gtggtgtgca gcgagaatag cttgcagttc ttcatgcccg tgttgggtgc

781 cctgttcatc ggtgtggctg tggccccagc taacgacatc tacaacgagc gcgagctgct

841 gaacagcatg ggcatcagcc agcccaccgt cgtattcgtg agcaagaaag ggctgcaaaa

901 gatcctcaac gtgcaaaaga agctaccgat catacaaaag atcatcatca tggatagcaa

961 gaccgactac cagggcttcc aaagcatgta caccttcgtg acttcccatt tgccacccgg

1021 cttcaacgag tacgacttcg tgcccgagag cttcgaccgg gacaaaacca tcgccctgat

1081 catgaacagt agtggcagta ccggattgcc caagggcgta gccctaccgc accgcaccgc

1141 ttgtgtccga ttcagtcatg cccgcgaccc catcttcggc aaccagatca tccccgacac

1201 cgctatcctc agcgtggtgc catttcacca cggcttcggc atgttcacca cgctgggcta

1261 cttgatctgc ggctttcggg tcgtgctcat gtaccgcttc gaggaggagc tattcttgcg

1321 cagcttgcaa gactataaga ttcaatctgc cctgctggtg cccacactat ttagcttctt

1381 cgctaagagc actctcatcg acaagtacga cctaagcaac ttgcacgaga tcgccagcgg

1441 cggggcgccg ctcagcaagg aggtaggtga ggccgtggcc aaacgcttcc acctaccagg

1501 catccgccag ggctacggcc tgacagaaac aaccagcgcc attctgatca cccccgaagg

1561 ggacgacaag cctggcgcag taggcaaggt ggtgcccttc ttcgaggcta aggtggtgga

1621 cttggacacc ggtaagacac tgggtgtgaa ccagcgcggc gagctgtgcg tccgtggccc

1681 catgatcatg agcggctacg ttaacaaccc cgaggctaca aacgctctca tcgacaagga

1741 cggctggctg cacagcggcg acatcgccta ctgggacgag gacgagcact tcttcatcgt

1801 ggaccggctg aagagcctga tcaaatacaa gggctaccag gtagccccag ccgaactgga

1861 gagcatcctg ctgcaacacc ccaacatctt cgacgccggg gtcgccggcc tgcccgacga

1921 cgatgccggc gagctgcccg ccgcagtcgt cgtgctggaa cacggtaaaa ccatgaccga

1981 gaaggagatc gtggactatg tggccagcca ggttacaacc gccaagaagc tgcgcggtgg

2041 tgttgtgttc gtggacgagg tgcctaaagg actgaccggc aagttggacg cccgcaagat

2101 ccgcgagatt ctcattaagg ccaagaaggg cggcaagatc gccgtgtaat gatagggtac

2161 cttgtttatt gcagcttata atggttacaa ataaagcaat agcatcacaa atttcacaaa

2221 taaagcattt ttttcactgc attctagttg tggtttgtcc aaactcatca atgtatctta

2281 tgcatgaaga atctgcttag ggttaggcgt tttgcgctgc ttcgcgatgt acgggccaga

2341 tatacgcgtt gacattgatt attgactagt tattaatagt aatcaattac ggggtcatta

2401 gttcatagcc catatatgga gttccgcgtt acataactta cggtaaatgg cccgcctggc

2461 tgaccgccca acgacccccg cccattgacg tcaataatga cgtatgttcc catagtaacg

2521 ccaataggga ctttccattg acgtcaatgg gtggagtatt tacggtaaac tgcccacttg

2581 gcagtacatc aagtgtatca tatgccaagt acgcccccta ttgacgtcaa tgacggtaaa

2641 tggcccgcct ggcattatgc ccagtacatg accttatggg actttcctac ttggcagtac

2701 atctacgtat tagtcatcgc tattaccatg gtgatgcggt tttggcagta catcaatggg

2761 cgtggatagc ggtttgactc acggggattt ccaagtctcc accccattga cgtcaatggg

2821 agtttgtttt ggcaccaaaa tcaacgggac tttccaaaat gtcgtaacaa ctccgcccca

2881 ttgacgcaaa tgggcggtag gcgtgtacgg tgggaggtct atataagcag agctctctgg

2941 ctaactagag aacccactgc ttactggctt atcgaaatta atacgactca ctatagggag

3001 acccaagctg gctagcgttt aaacttaagc ttgccaccat ggtattcaca ctcgaagatt

3061 tcgttgggga ctggcgacag acagccggct acaacctgga ccaagtcctt gaacagggag

3121 gtgtgtccag tttgtttcag aatctcgggg tgtccgtaac tccgatccaa aggattgtcc

3181 tgagcggtga aaatgggctg aagatcgaca tccatgtcat catcccgtat gaaggtctga

3241 gcggcgacca aatgggccag atcgaaaaaa tttttaaggt ggtgtaccct gtggatgatc

3301 atcactttaa ggtgatcctg cactatggca cactggtaat cgacggggtt acgccgaaca

3361 tgatcgacta tttcggacgg ccgtatgaag gcatcgccgt gttcgacggc aaaaagatca

3421 ctgtaacagg gaccctgtgg aacggcaaca aaattatcga cgagcgcctg atcaaccccg

3481 acggctccct gctgttccga gtaaccatca acggatgagt gaccggctgg cggctgtgcg

3541 aacgcattct ggcgtaagaa ttctaagggc gaattctgca gatatccagc acagtggcgg

3601 ccgctcgagt ctagattgtt tattgcagct tataatggtt acaaataaag caatagcatc

3661 acaaatttca caaataaagc atttttttca ctgcattcta gttgtggttt gtccaaactc

3721 atcaatgtat cttatcatgt ctggatcgct agagggcctt cctaatacga ctcactatag

3781 agcgctccgg tttttctgtg ctgaacctca ggggacgccg acacacgtac acgtctagtc

3841 ttcgcggccg cattaggcac cccaggcttt acactttatg cttccggctc gtataatgtg

3901 tggattttga gttaggatcc gtcgagattt tcaggagcta aggaagctaa aatggagaaa

3961 aaaatcactg gatataccac cgttgatata tcccaatggc atcgtaaaga acattttgag

4021 gcatttcagt cagttgctca atgtacctat aaccagaccg ttcagctgga tattacggcc

4081 tttttaaaga ccgtaaagaa aaataagcac aagttttatc cggcctttat tcacattctt

4141 gcccgcctga tgaatgctca tccggaattc cgtatggcaa tgaaagacgg tgagctggtg

4201 atatgggata gtgttcaccc ttgttacacc gttttccatg agcaaactga aacgttttca

4261 tcgctctgga gtgaatacca cgacgatttc cggcagtttc tacacatata ttcgcaagat

4321 gtggcgtgtt acggtgaaaa cctggcctat ttccctaaag ggtttattga gaatatgttt

4381 ttcgtctcag ccaatccctg ggtgagtttc accagttttg atttaaacgt ggccaatatg

4441 gacaacttct tcgcccccgt tttcaccatg ggcaaatatt atacgcaagg cgacaaggtg

4501 ctgatgccgc tggcgattca ggttcatcat gccgtttgtg atggcttcca tgtcggcaga

4561 atgcttaatg aattacaaca gtactgcgat gagtggcagg gcggggcgta aagatctgga

4621 tccggcttac taaaagccag ataacagtat gcgtatttgc gcgctgattt ttgcggtata

4681 agaatatata ctgatatgta tacccgaagt atgtcaaaaa gaggtatgct atgaagcagc

4741 gtattacagt gacagttgac agcgacagct atcagttgct caaggcatat atgatgtcaa

4801 tatctccggt ctggtaagca caaccatgca gaatgaagcc cgtcgtctgc gtgccgaacg

4861 ctggaaagcg gaaaatcagg aagggatggc tgaggtcgcc cggtttattg aaatgaacgg

4921 ctcttttgct gacgagaaca ggggctggtg aaatgcagtt taaggtttac acctataaaa

4981 gagagagccg ttatcgtctg tttgtggatg tacagagtga tattattgac acgcccgggc

5041 gacggatggt gatccccctg gccagtgcac gtctgctgtc agataaagtc tcccgtgaac

5101 tttacccggt ggtgcatatc ggggatgaaa gctggcgcat gatgaccacc gatatggcca

5161 gtgtgcccgt ctccgttatc ggggaagaag tggctgatct cagccaccgc gaaaatgaca

5221 tcaaaaacgc cattaacctg atgttctggg gaatataaat gtcaggctcc cttatacaca

5281 gccagtctgc agggaagaag accggtcctt tttttgcttt agtgagggtt aattcaggca

5341 tgtgagcaaa aggccagcaa aaggccagga accgtaaaaa ggccgcgttg ctggcgtttt

5401 tccataggct ccgcccccct gacgagcatc acaaaaatcg acgctcaagt cagaggtggc

5461 gaaacccgac aggactataa agataccagg cgtttccccc tggaagctcc ctcgtgcgct

5521 ctcctgttcc gaccctgccg cttaccggat acctgtccgc ctttctccct tcgggaagcg

5581 tggcgctttc tcaatgctca cgctgtaggt atctcagttc ggtgtaggtc gttcgctcca

5641 agctgggctg tgtgcacgaa ccccccgttc agcccgaccg ctgcgcctta tccggtaact

5701 atcgtcttga gtccaacccg gtaagacacg acttatcgcc actggcagca gccactggta

5761 acaggattag cagagcgagg tatgtaggcg gtgctacaga gttcttgaag tggtggccta

5821 actacggcta cactagaagg acagtatttg gtatctgcgc tctgctgaag ccagttacct

5881 tcggaaaaag agttggtagc tcttgatccg gcaaacaaac caccgctacc agcggtggtt

5941 tttttgtttg caagcagcag attacgcgca gaaaaaaagg atctcaagaa gatcctttga

6001 tcttttctac ggggtctgac gctcagtgga acgaaaactc acgttaaggg attttggtca

6061 tgagattatc aaaaaggatc ttcacctaga tccttttaaa ttaaaaatga agttttaaat

6121 caatctaaag tatatatgag taaacttggt ctgacagtta ccaatgctta atcagtgagg

6181 cacctatctc agcgatctgt ctatttcgtt catccatagt tgcctgactc cccgtcgtgt

6241 agataactac gatacgggag ggcttaccat ctggccccag tgctgcaatg ataccgcgag

6301 acccacgctc accggctcca gatttatcag caataaacca gccagccgga agggccgagc

6361 gcagaagtgg tcctgcaact ttatccgcct ccatccagtc tattaattgt tgccgggaag

6421 ctagagtaag tagttcgcca gttaatagtt tgcgcaacgt tgttgccatt gctacaggca

6481 tcgtggtgtc acgctcgtcg tttggtatgg cttcattcag ctccggttcc caacgatcaa

6541 ggcgagttac atgatccccc atgttgtgca aaaaagcggt tagctccttc ggtcctccga

6601 tcgttgtcag aagtaagttg gccgcagtgt tatcactcat ggttatggca gcactgcata

6661 attctcttac tgtcatgcca tccgtaagat gcttttctgt gactggtgag tactcaacca

6721 agtcattctg agaatagtgt atgcggcgac cgagttgctc ttgcccggcg tcaatacggg

6781 ataataccgc gccacatagc agaactttaa aagtgctcat cattggaaaa cgttcttcgg

6841 ggcgaaaact ctcaaggatc ttaccgctgt tgagatccag ttcgatgtaa cccactcgtg

6901 cacccaactg atcttcagca tcttttactt tcaccagcgt ttctgggtga gcaaaaacag

6961 gaaggcaaaa tgccgcaaaa aagggaataa gggcgacacg gaaatgttga atactcatac

7021 tcttcctttt tcaatattat tgaagcattt atcagggtta ttgtctcatg agcggataca

7081 tatttgaatg tatttagaaa aataaacaaa taggggttcc gcgcacattt ccccgaaaag

7141 tgccac

//

**Genbank sequence for sfGFP-TGA-150 pcDNA3.1 Hygro(+)**

LOCUS sfGFP-TGA-150_pc 6322 bp DNA circular SYN 29-MAR-2023

DEFINITION pcDNA3.1/Hygro(+).

ACCESSION .

VERSION .

KEYWORDS .

SOURCE synthetic DNA construct

ORGANISM synthetic DNA construct

REFERENCE 1 (bases 1 to 6322)

AUTHORS Lueck

TITLE Direct Submission

JOURNAL Exported Mar 29, 2023 from SnapGene 6.2.1

https://www.snapgene.com

FEATURES Location/Qualifiers

source 1..6322

/mol_type="other DNA"

/organism="pcDNA3.1/Hygro(+)"

enhancer 235..614

/label=CMV enhancer

/note="human cytomegalovirus immediate early enhancer"

promoter 615..818

/label=CMV promoter

/note="human cytomegalovirus (CMV) immediate early

promoter"

promoter 863..881

/label=T7 promoter

/note="promoter for bacteriophage T7 RNA polymerase"

regulatory 903..912

/label=Kozak sequence

/note="vertebrate consensus sequence for strong initiation

of translation (Kozak, 1987)"

/regulatory_class="other"

CDS 909..1625

/codon_start=1

/product="GFP variant that folds robustly even when fused

to poorly folded proteins (Pedelacq et al., 2006)"

/label=superfolder GFP

/note="mammalian codon-optimized"

/translation="MVSKGEELFTGVVPILVELDGDVNGHKFSVRGEGEGDATNGKLTL

KFICTTGKLPVPWPTLVTTLTYGVQCFSRYPDHMKRHDFFKSAMPEGYVQERTISFKDD

GTYKTRAEVKFEGDTLVNRIELKGIDFKEDGNILGHKLEYNFNSH*VYITADKQKNGIK

ANFKIRHNVEDGSVQLADHYQQNTPIGDGPVLLPDNHYLSTQSVLSKDPNEKRDHMVLL

EFVTAAGITHGMDELYK"

misc_feature 1356..1358

/label=TGA150

CDS 1635..1658

/codon_start=1

/product="peptide that binds Strep-Tactin(R), an engineered

form of streptavidin"

/label=Strep-Tag II

/translation="WSHPQFEK"

CDS 1662..1685

/codon_start=1

/product="8xHis affinity tag"

/label=8xHis

/translation="HHHHHHHH"

CDS 1701..1724

/codon_start=1

/product="peptide that binds Strep-Tactin(R), an engineered

form of streptavidin"

/label=Strep-Tag II

/translation="WSHPQFEK"

polyA_signal 1753..1977

/label=bGH poly(A) signal

/note="bovine growth hormone polyadenylation signal"

rep_origin 2023..2451

/direction=RIGHT

/label=f1 ori

/note="f1 bacteriophage origin of replication; arrow

indicates direction of (+) strand synthesis"

promoter 2465..2794

/label=SV40 promoter

/note="SV40 enhancer and early promoter"

rep_origin 2645..2780

/label=SV40 ori

/note="SV40 origin of replication"

CDS 2843..3868

/codon_start=1

/gene="aph(4)-Ia"

/product="aminoglycoside phosphotransferase from E. coli"

/label=HygR

/note="confers resistance to hygromycin"

/translation="MKKPELTATSVEKFLIEKFDSVSDLMQLSEGEESRAFSFDVGGRG

YVLRVNSCADGFYKDRYVYRHFASAALPIPEVLDIGEFSESLTYCISRRAQGVTLQDLP

ETELPAVLQPVAEAMDAIAAADLSQTSGFGPFGPQGIGQYTTWRDFICAIADPHVYHWQ

TVMDDTVSASVAQALDELMLWAEDCPEVRHLVHADFGSNNVLTDNGRITAVIDWSEAMF

GDSQYEVANIFFWRPWLACMEQQTRYFERRHPELAGSPRLRAYMLRIGLDQLYQSLVDG

NFDDAAWAQGRCDAIVRSGAGTVGRTQIARRSAAVWTDGCVEVLADSGNRRPSTRPRAK

E"

polyA_signal 3998..4119

/label=SV40 poly(A) signal

/note="SV40 polyadenylation signal"

primer_bind complement(4168..4184)

/label=M13 rev

/note="common sequencing primer, one of multiple similar

variants"

protein_bind 4192..4208

/label=lac operator

/bound_moiety="lac repressor encoded by lacI"

/note="The lac repressor binds to the lac operator to

inhibit transcription in E. coli. This inhibition can be

relieved by adding lactose or

isopropyl-beta-D-thiogalactopyranoside (IPTG)."

promoter complement(4216..4246)

/label=lac promoter

/note="promoter for the E. coli lac operon"

protein_bind 4261..4282

/label=CAP binding site

/bound_moiety="E. coli catabolite activator protein"

/note="CAP binding activates transcription in the presence

of cAMP."

rep_origin complement(4570..5155)

/direction=LEFT

/label=ori

/note="high-copy-number ColE1/pMB1/pBR322/pUC origin of

replication"

CDS complement(5326..6186)

/codon_start=1

/gene="bla"

/product="beta-lactamase"

/label=AmpR

/note="confers resistance to ampicillin, carbenicillin, and

related antibiotics"

/translation="MSIQHFRVALIPFFAAFCLPVFAHPETLVKVKDAEDQLGARVGYI

ELDLNSGKILESFRPEERFPMMSTFKVLLCGAVLSRIDAGQEQLGRRIHYSQNDLVEYS

PVTEKHLTDGMTVRELCSAAITMSDNTAANLLLTTIGGPKELTAFLHNMGDHVTRLDRW

EPELNEAIPNDERDTTMPVAMATTLRKLLTGELLTLASRQQLIDWMEADKVAGPLLRSA

LPAGWFIADKSGAGERGSRGIIAALGPDGKPSRIVVIYTTGSQATMDERNRQIAEIGAS

LIKHW"

promoter complement(6187..6291)

/gene="bla"

/label=AmpR promoter

ORIGIN

1 gacggatcgg gagatctccc gatcccctat ggtgcactct cagtacaatc tgctctgatg

61 ccgcatagtt aagccagtat ctgctccctg cttgtgtgtt ggaggtcgct gagtagtgcg

121 cgagcaaaat ttaagctaca acaaggcaag gcttgaccga caattgcatg aagaatctgc

181 ttagggttag gcgttttgcg ctgcttcgcg atgtacgggc cagatatacg cgttgacatt

241 gattattgac tagttattaa tagtaatcaa ttacggggtc attagttcat agcccatata

301 tggagttccg cgttacataa cttacggtaa atggcccgcc tggctgaccg cccaacgacc

361 cccgcccatt gacgtcaata atgacgtatg ttcccatagt aacgccaata gggactttcc

421 attgacgtca atgggtggag tatttacggt aaactgccca cttggcagta catcaagtgt

481 atcatatgcc aagtacgccc cctattgacg tcaatgacgg taaatggccc gcctggcatt

541 atgcccagta catgacctta tgggactttc ctacttggca gtacatctac gtattagtca

601 tcgctattac catggtgatg cggttttggc agtacatcaa tgggcgtgga tagcggtttg

661 actcacgggg atttccaagt ctccacccca ttgacgtcaa tgggagtttg ttttggcacc

721 aaaatcaacg ggactttcca aaatgtcgta acaactccgc cccattgacg caaatgggcg

781 gtaggcgtgt acggtgggag gtctatataa gcagagctct ctggctaact agagaaccca

841 ctgcttactg gcttatcgaa attaatacga ctcactatag ggacacccaa gctggctagg

901 ccgccaccat ggtttccaaa ggagaagagt tgttcacagg tgtcgttcct atccttgtcg

961 agctggacgg tgatgtgaat ggacacaagt ttagtgtcag gggggaagga gagggcgatg

1021 ctaccaacgg gaaactgacc ctgaaattca tttgtacaac gggtaagctg cctgttcctt

1081 ggcccacgct ggtcacgacg cttacctatg gcgtgcaatg cttcagtcgc tatcccgatc

1141 atatgaaaag acatgacttt ttcaagtccg caatgccaga aggctacgtg caggagagaa

1201 caatcagctt taaggacgat ggcacgtaca aaactcgggc cgaggtcaag tttgagggag

1261 acacattggt aaacagaatt gagctgaagg ggatcgactt taaggaagac ggtaatattc

1321 tcggacacaa gttggagtat aacttcaatt cacactgagt gtatataact gccgataagc

1381 aaaaaaatgg tatcaaggct aattttaaaa tccggcacaa tgtagaagac ggctccgtgc

1441 aactggcgga tcactaccag cagaacaccc ccatcggcga tggtccagtt ttgctgcccg

1501 ataatcatta tctcagcacc cagagcgtgc tttctaaaga tccaaatgaa aagagggatc

1561 acatggtcct tttggagttt gttacggctg ccggaatcac ccacgggatg gacgagctct

1621 acaagggcgg aagttggagc catccgcagt ttgaaaaagc gcaccaccat caccatcatc

1681 atcactccgg gggcagtgca tggtcacacc ctcagtttga gaagtaatag tgaacccgct

1741 gatcagcctc gactgtgcct tctagttgcc agccatctgt tgtttgcccc tcccccgtgc

1801 cttccttgac cctggaaggt gccactccca ctgtcctttc ctaataaaat gaggaaattg

1861 catcgcattg tctgagtagg tgtcattcta ttctgggggg tggggtgggg caggacagca

1921 agggggagga ttgggaagac aatagcaggc atgctgggga tgcggtgggc tctatggctt

1981 ctgaggcgga aagaaccagc tggggctcta gggggtatcc ccacgcgccc tgtagcggcg

2041 cattaagcgc ggcgggtgtg gtggttacgc gcagcgtgac cgctacactt gccagcgccc

2101 tagcgcccgc tcctttcgct ttcttccctt cctttctcgc cacgttcgcc ggctttcccc

2161 gtcaagctct aaatcggggg ctccctttag ggttccgatt tagtgcttta cggcacctcg

2221 accccaaaaa acttgattag ggtgatggtt cacgtagtgg gccatcgccc tgatagacgg

2281 tttttcgccc tttgacgttg gagtccacgt tctttaatag tggactcttg ttccaaactg

2341 gaacaacact caaccctatc tcggtctatt cttttgattt ataagggatt ttgccgattt

2401 cggcctattg gttaaaaaat gagctgattt aacaaaaatt taacgcgaat taattctgtg

2461 gaatgtgtgt cagttagggt gtggaaagtc cccaggctcc ccagcaggca gaagtatgca

2521 aagcatgcat ctcaattagt cagcaaccag gtgtggaaag tccccaggct ccccagcagg

2581 cagaagtatg caaagcatgc atctcaatta gtcagcaacc atagtcccgc ccctaactcc

2641 gcccatcccg cccctaactc cgcccagttc cgcccattct ccgccccatg gctgactaat

2701 tttttttatt tatgcagagg ccgaggccgc ctctgcctct gagctattcc agaagtagtg

2761 aggaggcttt tttggaggcc taggcttttg caaaaagctc ccgggagctt gtatatccat

2821 tttcggatct gatcagcacg tgatgaaaaa gcctgaactc accgcgacgt ctgtcgagaa

2881 gtttctgatc gaaaagttcg acagcgtctc cgacctgatg cagctctcgg agggcgaaga

2941 atctcgtgct ttcagcttcg atgtaggagg gcgtggatat gtcctgcggg taaatagctg

3001 cgccgatggt ttctacaaag atcgttatgt ttatcggcac tttgcatcgg ccgcgctccc

3061 gattccggaa gtgcttgaca ttggggaatt cagcgagagc ctgacctatt gcatctcccg

3121 ccgtgcacag ggtgtcacgt tgcaagacct gcctgaaacc gaactgcccg ctgttctgca

3181 gccggtcgcg gaggccatgg atgcgatcgc tgcggccgat cttagccaga cgagcgggtt

3241 cggcccattc ggaccgcaag gaatcggtca atacactaca tggcgtgatt tcatatgcgc

3301 gattgctgat ccccatgtgt atcactggca aactgtgatg gacgacaccg tcagtgcgtc

3361 cgtcgcgcag gctctcgatg agctgatgct ttgggccgag gactgccccg aagtccggca

3421 cctcgtgcac gcggatttcg gctccaacaa tgtcctgacg gacaatggcc gcataacagc

3481 ggtcattgac tggagcgagg cgatgttcgg ggattcccaa tacgaggtcg ccaacatctt

3541 cttctggagg ccgtggttgg cttgtatgga gcagcagacg cgctacttcg agcggaggca

3601 tccggagctt gcaggatcgc cgcggctccg ggcgtatatg ctccgcattg gtcttgacca

3661 actctatcag agcttggttg acggcaattt cgatgatgca gcttgggcgc agggtcgatg

3721 cgacgcaatc gtccgatccg gagccgggac tgtcgggcgt acacaaatcg cccgcagaag

3781 cgcggccgtc tggaccgatg gctgtgtaga agtactcgcc gatagtggaa accgacgccc

3841 cagcactcgt ccgagggcaa aggaatagca cgtgctacga gatttcgatt ccaccgccgc

3901 cttctatgaa aggttgggct tcggaatcgt tttccgggac gccggctgga tgatcctcca

3961 gcgcggggat ctcatgctgg agttcttcgc ccaccccaac ttgtttattg cagcttataa

4021 tggttacaaa taaagcaata gcatcacaaa tttcacaaat aaagcatttt tttcactgca

4081 ttctagttgt ggtttgtcca aactcatcaa tgtatcttat catgtctgta taccgtcgac

4141 ctctagctag agcttggcgt aatcatggtc atagctgttt cctgtgtgaa attgttatcc

4201 gctcacaatt ccacacaaca tacgagccgg aagcataaag tgtaaagcct ggggtgccta

4261 atgagtgagc taactcacat taattgcgtt gcgctcactg cccgctttcc agtcgggaaa

4321 cctgtcgtgc cagctgcatt aatgaatcgg ccaacgcgcg gggagaggcg gtttgcgtat

4381 tgggcgctct tccgcttcct cgctcactga ctcgctgcgc tcggtcgttc ggctgcggcg

4441 agcggtatca gctcactcaa aggcggtaat acggttatcc acagaatcag gggataacgc

4501 aggaaagaac atgtgagcaa aaggccagca aaaggccagg aaccgtaaaa aggccgcgtt

4561 gctggcgttt ttccataggc tccgcccccc tgacgagcat cacaaaaatc gacgctcaag

4621 tcagaggtgg cgaaacccga caggactata aagataccag gcgtttcccc ctggaagctc

4681 cctcgtgcgc tctcctgttc cgaccctgcc gcttaccgga tacctgtccg cctttctccc

4741 ttcgggaagc gtggcgcttt ctcatagctc acgctgtagg tatctcagtt cggtgtaggt

4801 cgttcgctcc aagctgggct gtgtgcacga accccccgtt cagcccgacc gctgcgcctt

4861 atccggtaac tatcgtcttg agtccaaccc ggtaagacac gacttatcgc cactggcagc

4921 agccactggt aacaggatta gcagagcgag gtatgtaggc ggtgctacag agttcttgaa

4981 gtggtggcct aactacggct acactagaag aacagtattt ggtatctgcg ctctgctgaa

5041 gccagttacc ttcggaaaaa gagttggtag ctcttgatcc ggcaaacaaa ccaccgctgg

5101 tagcggtttt tttgtttgca agcagcagat tacgcgcaga aaaaaaggat ctcaagaaga

5161 tcctttgatc ttttctacgg ggtctgacgc tcagtggaac gaaaactcac gttaagggat

5221 tttggtcatg agattatcaa aaaggatctt cacctagatc cttttaaatt aaaaatgaag

5281 ttttaaatca atctaaagta tatatgagta aacttggtct gacagttacc aatgcttaat

5341 cagtgaggca cctatctcag cgatctgtct atttcgttca tccatagttg cctgactccc

5401 cgtcgtgtag ataactacga tacgggaggg cttaccatct ggccccagtg ctgcaatgat

5461 accgcgagac ccacgctcac cggctccaga tttatcagca ataaaccagc cagccggaag

5521 ggccgagcgc agaagtggtc ctgcaacttt atccgcctcc atccagtcta ttaattgttg

5581 ccgggaagct agagtaagta gttcgccagt taatagtttg cgcaacgttg ttgccattgc

5641 tacaggcatc gtggtgtcac gctcgtcgtt tggtatggct tcattcagct ccggttccca

5701 acgatcaagg cgagttacat gatcccccat gttgtgcaaa aaagcggtta gctccttcgg

5761 tcctccgatc gttgtcagaa gtaagttggc cgcagtgtta tcactcatgg ttatggcagc

5821 actgcataat tctcttactg tcatgccatc cgtaagatgc ttttctgtga ctggtgagta

5881 ctcaaccaag tcattctgag aatagtgtat gcggcgaccg agttgctctt gcccggcgtc

5941 aatacgggat aataccgcgc cacatagcag aactttaaaa gtgctcatca ttggaaaacg

6001 ttcttcgggg cgaaaactct caaggatctt accgctgttg agatccagtt cgatgtaacc

6061 cactcgtgca cccaactgat cttcagcatc ttttactttc accagcgttt ctgggtgagc

6121 aaaaacagga aggcaaaatg ccgcaaaaaa gggaataagg gcgacacgga aatgttgaat

6181 actcatactc ttcctttttc aatattattg aagcatttat cagggttatt gtctcatgag

6241 cggatacata tttgaatgta tttagaaaaa taaacaaata ggggttccgc gcacatttcc

6301 ccgaaaagtg ccacctgacg tc

//

**Genbank sequence for 2xLeuUGA scAAV insert**

LOCUS 2xLeuUGA_insert 1948 bp DNA linear UNA 15-APR-2024

DEFINITION natural linear DNA

ACCESSION .

VERSION .

KEYWORDS .

SOURCE natural DNA sequence

ORGANISM unspecified

REFERENCE 1 (bases 1 to 1948)

AUTHORS Lueck

TITLE Direct Submission

JOURNAL Exported Apr 15, 2024 from SnapGene 7.2.0

https://www.snapgene.com

FEATURES Location/Qualifiers

source 1..1948

/mol_type="genomic DNA"

/organism="unspecified"

source 437..585

/lab_host="E.coli"

/db_xref="taxon:66198"

/organism="Cloning vector pUC57"

source 1387..1535

/lab_host="E.coli"

/db_xref="taxon:66198"

/organism="Cloning vector pUC57"

gene complement(437..585)

/gene="lacZ"

/label=lacZ

misc_feature 437..491

/label=tRNA Tyr Leader

misc_feature 492..574

/label=LeuTGAchr11.tRNA4

misc_feature 575..585

/label=RNA polIII term

primer_bind 962..979

/label=M13 Forward

/note="In lacZ gene. Also called M13-F20 or M13 (-21)

Forward"

primer_bind 963..979

/label=M13 fwd

/note="common sequencing primer, one of multiple similar

variants"

gene complement(1387..1535)

/gene="lacZ"

/label=lacZ

misc_feature 1387..1441

/label=tRNA Tyr Leader

misc_feature 1442..1524

/label=LeuTGAchr11.tRNA4

misc_feature 1525..1535

/label=RNA polIII term

ORIGIN

1 caattgggtc gacggtatcg ataagcttga taacgcttag ggataacagg gtaatcttaa

61 ggctcgacat gtccaacggt tgcccaaagt gttaagtgtc tatcacccct agggccgttt

121 cccggatata aacgccaggt tgaatccgca tttgaagcta ccatggatga gtctgggtcg

181 agcgcgccgc atttattgcg tgagtagggt cgaccaagaa ccgctagatg cgtcgctgta

241 caaatagttg tcgacagacc gtcgagttta gaaaatggta ccagcatttt cgggggatct

301 caatcaagta tggattacgg tgtttacact gtcctgcggc tacccatggc ctgaaatcca

361 gctcgtgtca agccattgcc tctccgggac gccgcatgaa gtaatacata taccttgcac

421 gggttcactg cggtccagcg ctccggtttt tctgtgctga acctcagggg acgccgacac

481 acgtacacgt caccagaatg gccgagtggt taaggcgttg gacttcagat ccaatggatt

541 catatccgcg tgggttcgaa ccccacttct ggtagtcctt tttttaagcc ccccgctcgg

601 gagtaccaga gaagatgtct actgagttgt gcgatccctg cacttcagct aaggaagcta

661 ccaatattta gtttctgagt ctcacgacag acctcgcgcg tagattgcca tgcgtagagc

721 taacgagcca gcggaaagcg tgaggcgctt ttaagcatgg cgagtaagtg atccaacgct

781 tcggatatga ctatatactt aggttcgatc tcgtcccgag aattctaagc ctcaacatct

841 atgagttatg aggttagccg aaaaagcacg tggtggcgcc caccgactgt tcccagactg

901 tagctctttg ttctgtcaag gcccgacctt catcgcggcc gattccttct gcggaggatc

961 ctgtaaaacg acggccagtg tattctagag caataaccca tcaatctgta cgcaccctca

1021 gatagtgggg atcccgggta tagaccttta tctgcggtcc aacttaggca taaacctcca

1081 tgctaccttg tcagacccac cctgcacgag gtaaatatgg gacgcgtccg acctggctcc

1141 tggcgttcta cgccgccacg tgttcgttaa ctgttgattg gtagcacaaa agtaatacca

1201 tggtccttga aattcggctc agttagttcg agcgtaatgt cacaaatggc gcagaacggc

1261 aatgagtgtt tgacactagg tggtgttcag ttcggtaacg gagagactgt gcggcatact

1321 taattataca tttgaaacgc gcccaagtga cgctaggcaa gtcagagcag gttcccgtgt

1381 tagcttagcg ctccggtttt tctgtgctga acctcagggg acgccgacac acgtacacgt

1441 caccagaatg gccgagtggt taaggcgttg gacttcagat ccaatggatt catatccgcg

1501 tgggttcgaa ccccacttct ggtagtcctt tttttagttg attcgaacga tggttataaa

1561 tcaaaaaaac ggaacgctgt ctggaggatg aatctaacgg tgcgtaactc gatcactcac

1621 tcgctattcg aactgcgcga aagttcccag cgctcataca cttggttccg aggcctgtcc

1681 tgatatatga acccaaacta gagcggggct gttgacgttt ggagttgaaa aaatctaata

1741 ttccaatcgg cttcaacgtg caccaccgca ggcggctgac gaggggctca caccgagaaa

1801 gtagactgtt gcgcgttggg ggtagcgccg gctaacaaag acgcctggta cagcaggagt

1861 atcaaacccg tacaaagctt cgccagggtt ttccaagtcg atcacctaca gattgctaga

1921 gtatgccttt ccagtctaga ttaattaa

//

**Genbank sequence for 2xOptLeuUGA scAAV insert**

LOCUS 2xOptLeuUGA_inse 1996 bp DNA linear UNA 15-APR-2024

DEFINITION natural linear DNA

ACCESSION .

VERSION .

KEYWORDS .

SOURCE natural DNA sequence

ORGANISM unspecified

REFERENCE 1 (bases 1 to 1996)

AUTHORS Lueck

TITLE Direct Submission

JOURNAL Exported Apr 15, 2024 from SnapGene 7.2.0

https://www.snapgene.com

FEATURES Location/Qualifiers

source 1..1996

/mol_type="genomic DNA"

/organism="unspecified"

source 492..574

/lab_host="E.coli"

/db_xref="taxon:66198"

/organism="Cloning vector pUC57"

source 1466..1548

/lab_host="E.coli"

/db_xref="taxon:66198"

/organism="Cloning vector pUC57"

misc_feature 437..491

/label=tRNA-Cys-GCA-12-1_F

gene complement(492..574)

/gene="lacZ"

/label=lacZ

misc_feature 492..574

/label=LeuTGAchr11.tRNA4 SS94 ts9

misc_feature 575..578

/label=4bp 3p

misc_feature 579..609

/label=tRNA-Ile-TAT-1-1

primer_bind 987..1003

/label=M13 fwd

/note="common sequencing primer, one of multiple similar

variants"

misc_feature 1411..1465

/label=tRNA-Cys-GCA-12-1_F

gene complement(1466..1548)

/gene="lacZ"

/label=lacZ

misc_feature 1466..1548

/label=LeuTGAchr11.tRNA4 SS94 ts9

misc_feature 1549..1552

/label=4bp 3p

misc_feature 1553..1583

/label=tRNA-Ile-TAT-1-1

ORIGIN

1 caattgggtc gacggtatcg ataagcttga taacgcttag ggataacagg gtaatcttaa

61 ggctcgacat gtccaacggt tgcccaaagt gttaagtgtc tatcacccct agggccgttt

121 cccggatata aacgccaggt tgaatccgca tttgaagcta ccatggatga gtctgggtcg

181 agcgcgccgc atttattgcg tgagtagggt cgaccaagaa ccgctagatg cgtcgctgta

241 caaatagttg tcgacagacc gtcgagttta gaaaatggta ccagcatttt cgggggatct

301 caatcaagta tggattacgg tgtttacact gtcctgcggc tacccatggc ctgaaatcca

361 gctcgtgtca agccattgcc tctccgggac gccgcatgaa gtaatacata taccttgcac

421 gggttcactg cggtccgcca ggggtgtggc catacaggtt tatagtggtt agtagagaca

481 agtaagtggg tgccaggatg gccgagtggt taaggcgccg gacttcagat ccggtggact

541 catgtccgcc cgggttcgaa ccccggtcct ggcagacctt ttccccatca gtttttataa

601 acttacacaa agccccccgc tcgggagtac cagagaagat gtctactgag ttgtgcgatc

661 cctgcacttc agctaaggaa gctaccaata tttagtttct gagtctcacg acagacctcg

721 cgcgtagatt gccatgcgta gagctaacga gccagcggaa agcgtgaggc gcttttaagc

781 atggcgagta agtgatccaa cgcttcggat atgactatat acttaggttc gatctcgtcc

841 cgagaattct aagcctcaac atctatgagt tatgaggtta gccgaaaaag cacgtggtgg

901 cgcccaccga ctgttcccag actgtagctc tttgttctgt caaggcccga ccttcatcgc

961 ggccgattcc ttctgcggag gatcctgtaa aacgacggcc agtgtattct agagcaataa

1021 cccatcaatc tgtacgcacc ctcagatagt ggggatcccg ggtatagacc tttatctgcg

1081 gtccaactta ggcataaacc tccatgctac cttgtcagac ccaccctgca cgaggtaaat

1141 atgggacgcg tccgacctgg ctcctggcgt tctacgccgc cacgtgttcg ttaactgttg

1201 attggtagca caaaagtaat accatggtcc ttgaaattcg gctcagttag ttcgagcgta

1261 atgtcacaaa tggcgcagaa cggcaatgag tgtttgacac taggtggtgt tcagttcggt

1321 aacggagaga ctgtgcggca tacttaatta tacatttgaa acgcgcccaa gtgacgctag

1381 gcaagtcaga gcaggttccc gtgttagctt gccaggggtg tggccataca ggtttatagt

1441 ggttagtaga gacaagtaag tgggtgccag gatggccgag tggttaaggc gccggacttc

1501 agatccggtg gactcatgtc cgcccgggtt cgaaccccgg tcctggcaga ccttttcccc

1561 atcagttttt ataaacttac acaagttgat tcgaacgatg gttataaatc aaaaaaacgg

1621 aacgctgtct ggaggatgaa tctaacggtg cgtaactcga tcactcactc gctattcgaa

1681 ctgcgcgaaa gttcccagcg ctcatacact tggttccgag gcctgtcctg atatatgaac

1741 ccaaactaga gcggggctgt tgacgtttgg agttgaaaaa atctaatatt ccaatcggct

1801 tcaacgtgca ccaccgcagg cggctgacga ggggctcaca ccgagaaagt agactgttgc

1861 gcgttggggg tagcgccggc taacaaagac gcctggtaca gcaggagtat caaacccgta

1921 caaagcttcg ccagggtttt ccaagtcgat cacctacaga ttgctagagt atgcctttcc

1981 agtctagatt aattaa

//

**Genbank sequence for 2xScrambled scAAV insert**

LOCUS 2xScram_insert 1948 bp DNA linear UNA 15-APR-2024

DEFINITION natural linear DNA

ACCESSION .

VERSION .

KEYWORDS .

SOURCE natural DNA sequence

ORGANISM unspecified

REFERENCE 1 (bases 1 to 1948)

AUTHORS Lueck

TITLE Direct Submission

JOURNAL Exported Apr 15, 2024 from SnapGene 7.2.0

https://www.snapgene.com

FEATURES Location/Qualifiers

source 1..1948

/mol_type="genomic DNA"

/organism="unspecified"

source 437..585

/lab_host="E.coli"

/db_xref="taxon:66198"

/organism="Cloning vector pUC57"

source 1387..1535

/lab_host="E.coli"

/db_xref="taxon:66198"

/organism="Cloning vector pUC57"

gene complement(437..585)

/gene="lacZ"

/label=lacZ

misc_feature 437..491

/label=tRNA Tyr Leader

misc_feature 492..574

/label=LeuTGAscrambled

misc_feature 575..585

/label=RNA polIII term

primer_bind 963..979

/label=M13 fwd

/note="common sequencing primer, one of multiple similar

variants"

gene complement(1387..1535)

/gene="lacZ"

/label=lacZ

misc_feature 1387..1441

/label=tRNA Tyr Leader

misc_feature 1442..1524

/label=LeuTGAscrambled

misc_feature 1525..1535

/label=RNA polIII term

ORIGIN

1 caattgggtc gacggtatcg ataagcttga taacgcttag ggataacagg gtaatcttaa

61 ggctcgacat gtccaacggt tgcccaaagt gttaagtgtc tatcacccct agggccgttt

121 cccggatata aacgccaggt tgaatccgca tttgaagcta ccatggatga gtctgggtcg

181 agcgcgccgc atttattgcg tgagtagggt cgaccaagaa ccgctagatg cgtcgctgta

241 caaatagttg tcgacagacc gtcgagttta gaaaatggta ccagcatttt cgggggatct

301 caatcaagta tggattacgg tgtttacact gtcctgcggc tacccatggc ctgaaatcca

361 gctcgtgtca agccattgcc tctccgggac gccgcatgaa gtaatacata taccttgcac

421 gggttcactg cggtccagcg ctccggtttt tctgtgctga acctcagggg acgccgacac

481 acgtacacgt cggttggaac ctatatgaca atccggaagc tgttcgtcta gattcgacga

541 gccggagtac gctgccgacg tagttcagtt ctacgtcctt tttttaagcc ccccgctcgg

601 gagtaccaga gaagatgtct actgagttgt gcgatccctg cacttcagct aaggaagcta

661 ccaatattta gtttctgagt ctcacgacag acctcgcgcg tagattgcca tgcgtagagc

721 taacgagcca gcggaaagcg tgaggcgctt ttaagcatgg cgagtaagtg atccaacgct

781 tcggatatga ctatatactt aggttcgatc tcgtcccgag aattctaagc ctcaacatct

841 atgagttatg aggttagccg aaaaagcacg tggtggcgcc caccgactgt tcccagactg

901 tagctctttg ttctgtcaag gcccgacctt catcgcggcc gattccttct gcggaggatc

961 ctgtaaaacg acggccagtg tattctagag caataaccca tcaatctgta cgcaccctca

1021 gatagtgggg atcccgggta tagaccttta tctgcggtcc aacttaggca taaacctcca

1081 tgctaccttg tcagacccac cctgcacgag gtaaatatgg gacgcgtccg acctggctcc

1141 tggcgttcta cgccgccacg tgttcgttaa ctgttgattg gtagcacaaa agtaatacca

1201 tggtccttga aattcggctc agttagttcg agcgtaatgt cacaaatggc gcagaacggc

1261 aatgagtgtt tgacactagg tggtgttcag ttcggtaacg gagagactgt gcggcatact

1321 taattataca tttgaaacgc gcccaagtga cgctaggcaa gtcagagcag gttcccgtgt

1381 tagcttagcg ctccggtttt tctgtgctga acctcagggg acgccgacac acgtacacgt

1441 cggttggaac ctatatgaca atccggaagc tgttcgtcta gattcgacga gccggagtac

1501 gctgccgacg tagttcagtt ctacgtcctt tttttagttg attcgaacga tggttataaa

1561 tcaaaaaaac ggaacgctgt ctggaggatg aatctaacgg tgcgtaactc gatcactcac

1621 tcgctattcg aactgcgcga aagttcccag cgctcataca cttggttccg aggcctgtcc

1681 tgatatatga acccaaacta gagcggggct gttgacgttt ggagttgaaa aaatctaata

1741 ttccaatcgg cttcaacgtg caccaccgca ggcggctgac gaggggctca caccgagaaa

1801 gtagactgtt gcgcgttggg ggtagcgccg gctaacaaag acgcctggta cagcaggagt

1861 atcaaacccg tacaaagctt cgccagggtt ttccaagtcg atcacctaca gattgctaga

1921 gtatgccttt ccagtctaga ttaattaa

//

**Supplemental References**

1. Goodenbour, J.M., and Pan, T. (2006). Diversity of tRNA genes in eukaryotes. Nucleic Acids Res *34*, 6137-6146. 10.1093/nar/gkl725.

2. Torres, A.G. (2019). Enjoy the Silence: Nearly Half of Human tRNA Genes Are Silent. Bioinform Biol Insights *13*, 1177932219868454. 10.1177/1177932219868454.

3. Morselli, M., and Dieci, G. (2022). Epigenetic regulation of human non-coding RNA gene transcription. Biochem Soc Trans *50*, 723-736. 10.1042/BST20210860.

4. Dieci, G., Fiorino, G., Castelnuovo, M., Teichmann, M., and Pagano, A. (2007). The expanding RNA polymerase III transcriptome. Trends Genet *23*, 614-622. 10.1016/j.tig.2007.09.001.

5. Galli, G., Hofstetter, H., and Birnstiel, M.L. (1981). Two conserved sequence blocks within eukaryotic tRNA genes are major promoter elements. Nature *294*, 626-631. 10.1038/294626a0.

6. Graczyk, D., Ciesla, M., and Boguta, M. (2018). Regulation of tRNA synthesis by the general transcription factors of RNA polymerase III - TFIIIB and TFIIIC, and by the MAF1 protein. Biochim Biophys Acta Gene Regul Mech *1861*, 320-329. 10.1016/j.bbagrm.2018.01.011.

7. Giuliodori, S., Percudani, R., Braglia, P., Ferrari, R., Guffanti, E., Ottonello, S., and Dieci, G. (2003). A composite upstream sequence motif potentiates tRNA gene transcription in yeast. J Mol Biol *333*, 1-20. 10.1016/j.jmb.2003.08.016.

8. Cabart, P., Lee, J., and Willis, I.M. (2008). Facilitated recycling protects human RNA polymerase III from repression by Maf1 in vitro. J Biol Chem *283*, 36108-36117. 10.1074/jbc.M807538200.

9. Bayfield, M.A., Yang, R., and Maraia, R.J. (2010). Conserved and divergent features of the structure and function of La and La-related proteins (LARPs). Biochim Biophys Acta *1799*, 365-378. 10.1016/j.bbagrm.2010.01.011.

10. Altman, S. (2007). A view of RNase P. Mol Biosyst *3*, 604-607. 10.1039/b707850c.

11. Schiffer, S., Rösch, S., and Marchfelder, A. (2002). Assigning a function to a conserved group of proteins: the tRNA 3'-processing enzymes. Embo j *21*, 2769-2777. 10.1093/emboj/21.11.2769.

12. Maraia, R.J., and Lamichhane, T.N. (2011). 3' processing of eukaryotic precursor tRNAs. Wiley Interdiscip Rev RNA *2*, 362-375. 10.1002/wrna.64.

13. Sun, L., Campbell, F.E., Zahler, N.H., and Harris, M.E. (2006). Evidence that substrate-specific effects of C5 protein lead to uniformity in binding and catalysis by RNase P. Embo j *25*, 3998-4007. 10.1038/sj.emboj.7601290.

14. Hou, Y.M. (2010). CCA addition to tRNA: implications for tRNA quality control. IUBMB Life *62*, 251-260. 10.1002/iub.301.

15. Gerber, J.L., Kohler, S., and Peschek, J. (2022). Eukaryotic tRNA splicing - one goal, two strategies, many players. Biol Chem *403*, 765-778. 10.1515/hsz-2021-0402.

16. Phizicky, E.M., and Alfonzo, J.D. (2010). Do all modifications benefit all tRNAs? FEBS Lett *584*, 265-271. 10.1016/j.febslet.2009.11.049.

17. Phizicky, E.M., and Hopper, A.K. (2010). tRNA biology charges to the front. Genes Dev *24*, 1832-1860. 10.1101/gad.1956510.

18. Giegé, R., and Eriani, G. (2021). Transfer RNA Recognition and Aminoacylation by Synthetases. In Encyclopedia of Life Sciences, (Wiley). 10.1002/9780470015902.a0029242.

19. Ibba, M., and Soll, D. (2000). Aminoacyl-tRNA synthesis. Annu Rev Biochem *69*, 617-650. 10.1146/annurev.biochem.69.1.617.

20. Janiak, F., Dell, V.A., Abrahamson, J.K., Watson, B.S., Miller, D.L., and Johnson, A.E. (1990). Fluorescence characterization of the interaction of various transfer RNA species with elongation factor Tu.cntdot.GTP: evidence for a new functional role for elongation factor Tu in protein biosynthesis. Biochemistry *29*, 4268-4277. 10.1021/bi00470a002.

21. Harvey, K.L., Jarocki, V.M., Charles, I.G., and Djordjevic, S.P. (2019). The Diverse Functional Roles of Elongation Factor Tu (EF-Tu) in Microbial Pathogenesis. Front Microbiol *10*, 2351. 10.3389/fmicb.2019.02351.

22. Sanderson, L.E., and Uhlenbeck, O.C. (2007). The 51-63 base pair of tRNA confers specificity for binding by EF-Tu. Rna *13*, 835-840. 10.1261/rna.485307.

23. Saks, M.E., Sanderson, L.E., Choi, D.S., Crosby, C.M., and Uhlenbeck, O.C. (2011). Functional consequences of T-stem mutations in E. coli tRNAThrUGU in vitro and in vivo. RNA *17*, 1038-1047. 10.1261/rna.2427311.

24. Uhlenbeck, O.C., and Schrader, J.M. (2018). Evolutionary tuning impacts the design of bacterial tRNAs for the incorporation of unnatural amino acids by ribosomes. Curr Opin Chem Biol *46*, 138-145. 10.1016/j.cbpa.2018.07.016.

25. Cochella, L., and Green, R. (2005). An active role for tRNA in decoding beyond codon:anticodon pairing. Science *308*, 1178-1180. 10.1126/science.1111408.

26. Moazed, D., and Noller, H.F. (1989). Interaction of tRNA with 23S rRNA in the ribosomal A, P, and E sites. Cell *57*, 585-597. 10.1016/0092-8674(89)90128-1.

27. Schmeing, T.M., Voorhees, R.M., Kelley, A.C., and Ramakrishnan, V. (2011). How mutations in tRNA distant from the anticodon affect the fidelity of decoding. Nat Struct Mol Biol *18*, 432-436. 10.1038/nsmb.2003.

28. Berg, M.D., and Brandl, C.J. (2021). Transfer RNAs: diversity in form and function. RNA Biol *18*, 316-339. 10.1080/15476286.2020.1809197.

29. Hopper, A.K. (2013). Transfer RNA post-transcriptional processing, turnover, and subcellular dynamics in the yeast Saccharomyces cerevisiae. Genetics *194*, 43-67. 10.1534/genetics.112.147470.

30. Guzzi, N., and Bellodi, C. (2020). Novel insights into the emerging roles of tRNA-derived fragments in mammalian development. RNA Biol *17*, 1214-1222. 10.1080/15476286.2020.1732694.

31. Akiyama, Y., and Ivanov, P. (2023). tRNA-derived RNAs: Biogenesis and roles in translational control. Wiley Interdiscip Rev RNA, e1805. 10.1002/wrna.1805.

32. Giege, R., and Eriani, G. (2023). The tRNA identity landscape for aminoacylation and beyond. Nucleic Acids Res *51*, 1528-1570. 10.1093/nar/gkad007.
